# Thermomagnetic properties and its effects on Fisher entropy with Schioberg plus Manning-Rosen potential (SPMRP) using Nikiforov-Uvarov functional analysis (NUFA) and supersymmetric quantum mechanics (SUSYQM) methods

**DOI:** 10.1038/s41598-023-34521-0

**Published:** 2023-05-20

**Authors:** I. B. Okon, C. A. Onate, R. Horchani, O. O. Popoola, E. Omugbe, E. S. William, U. S. Okorie, E. P. Inyang, C. N. Isonguyo, M. E. Udoh, A. D. Antia, W. L. Chen, E. S. Eyube, J. P. Araujo, A. N. Ikot

**Affiliations:** 1 Theoretical Physics Group, Department of Physics, University of Uyo, Uyo, Nigeria; 2 Department of Physics, Kogi State University, Anyigba, Nigeria; 3 Department of Physics, College of Science, Sultan Qaboos University, Muscat, Sultanate of Oman; 4 Department of Physics, University of Ibadan, Ibadan, Nigeria; 5 Department of Physics, University of Agriculture and Environmental Sciences, Umuagwo, P.M.B. 1038, Imo State Nigeria; 6 Department of Physics, Federal University of Technology, Ikot Abasi, Nigeria; 7 Department of Physics, Akwa Ibom State University, Mkpat-Enin, Nigeria; 8 Department of Physics, National Open University of Nigeria, Jabi-Abuja, Nigeria; 9 School of Intelligent Science and Information Engineering, Xi’an Peihua University, Xi’an, 710125 China; 10 Department of Physics, Faculty of Physical Sciences, Modibbo Adama University, P.M.B. 2076, Yola, Adamawa State Nigeria; 11 Department of Mathematics, Instituto Federal de Educação, Ciência e Tecnologia do Sudeste de Minas Gerais, Juiz de Fora, Brazil; 12 Theoretical Physics Group, Department of Physics, University of Port Harcourt, Port Harcourt, Nigeria

**Keywords:** Atomic and molecular physics, Chemical physics, Statistical physics, thermodynamics and nonlinear dynamics

## Abstract

Thermomagnetic properties, and its effects on Fisher information entropy with Schioberg plus Manning-Rosen potential are studied using NUFA and SUSYQM methods in the presence of the Greene-Aldrich approximation scheme to the centrifugal term. The wave function obtained was used to study Fisher information both in position and momentum spaces for different quantum states by the gamma function and digamma polynomials. The energy equation obtained in a closed form was used to deduce numerical energy spectra, partition function, and other thermomagnetic properties. The results show that with an application of AB and magnetic fields, the numerical energy eigenvalues for different magnetic quantum spins decrease as the quantum state increases and completely removes the degeneracy of the energy spectra. Also, the numerical computation of Fisher information satisfies Fisher information inequality products, indicating that the particles are more localized in the presence of external fields than in their absence, and the trend shows complete localization of quantum mechanical particles in all quantum states. Our potential reduces to Schioberg and Manning-Rosen potentials as special cases. Our potential reduces to Schioberg and Manning-Rosen potentials as special cases. The energy equations obtained from the NUFA and SUSYQM were the same, demonstrating a high level of mathematical precision.

## Introduction

Over the years, devices that enable quantum information to be coherently transferred between topological and conventional materials have been studied by various researchers^1^. These materials contained an electromagnetic field, which serves as a fundamental carrier of information, capable of transmitting a modulated signal and collecting data about the propagation channel itself^2^. This was made possible by the foundations of information theory by Fisher^3^ in his classical measurement theory, which he used for estimating ultimate quantum limits that allow for known local changes in density^4^. The context was also examined by Shannon^5^ in his study. The Shannon entropy is a global measure of electron density that plays a significant role in the assessment of uncertainty and provides a source of information about atomic, molecular, and nuclear systems^6–8^.

These theoretic tools provide a deeper understanding of density functional and electron correlation in studying the structure and dynamics of the atomic system^9^. Quantum information theory has proven to be extremely useful in a wide range of fields, such as Physics, Chemistry, Biology, Medicine, Computer science, neural networks, linguistics, and other social sciences^10^. They are commonly used in quantum physics to analyze quantum steering^11^, quantum entanglement^12^, quantum revivals^13^, quantum communication^14^, atomic ionization properties^15^, and other phenomena. In wave mechanics, the solutions of the eigenfunctions of the Schrödinger equation under a potential energy barrier are essential because the entropic functionals are presented in terms of probability densities in the position and momentum spaces^16^. Several research have been carried out on Shannon entropy and Fisher information with physically motivated potential models, like the class of Yukawa potential^17^, Screened Coulomb potential^9^, generalized hyperbolic potential^18^, screened Kratzer potential^19^, Frost-Musulin potential^20^, hyperbolic potential^21^, and many others.

The Manning-Rosen potential is a significant exponential-type potential proposed by Manning and Rosen^22^ in 1933 to explain the vibrational behavior of the model of the diatomic molecule^23^. The form of this potential model is given by^24^

1 $$V\left( r \right) = - \frac{{c_{1} e^{ - 2\alpha r} + c_{2} e^{ - \alpha r} }}{{\left( {1 - e^{ - \alpha r} } \right)^{2} }},$$

where $c_{1} {\text{ and }}c_{2}$ are potential strength parameters, and $\alpha$ represents the screening parameter while $r$ is the inter-particle distance. The Schioberg potential, proposed in 1986, is another intriguing potential. This potential describes the molecular vibrations of diatomic molecules accurately more than the Morse function and represents suitably intermolecular interactions between particles^25^. The potential model is of the form^26^

2 $$V\left( r \right) = D\left[ {1 - \delta_{0} \left( {\frac{{1 + e^{ - \alpha r} }}{{1 - e^{ - \alpha r} }}} \right)} \right]^{2} ,$$

where $D$ is the potential depth, $\alpha$ is the screening parameter and $\delta_{0}$ is the potential parameter that determines the size of the potential and can also serve as optimizing parameter. Recently, there has been a surge of interest in incorporating at least two potentials. The goal of combining at least two potential models is to provide more physical application and analysis to existing molecular physics studies. Also, it is well-known that the potential energy functions with more parameters have a tendency to fit experimental data better than those with fewer parameters^27–29^. Many scholars have conducted extensive research in both relativistic and non-relativistic regimes to explore these potentials^30–38^.

In recent times, research indicates that the addition of external fields to potential functions on quantum systems has demonstrated its potency in controlling certain behaviors of systems and molecules^39^. The Aharonov–Bohm (AB) effect, discovered in 1961^40^, occurs when a moving charge is transformed by scalar and vector potentials that appear in the Schrödinger equation (SE) even in the absence of external EM fields^41^. Since then, many studies have analyzed a bound state of a charged particle moving in a potential vector and scalar potential. A realistic description of the external EM field effects on quantum systems is provided by the Stark^42^ and Zeeman^43^ effects. In the Stark effects, an external electric field is applied to the electrically neutral hydrogen atom, causing it to experience a zero net force, resulting in a shift in the energy levels. On the other hand, Zeeman effects occur when an atom is exposed to a uniform magnetic field. These interactions have similar effects in that they cause the energy levels to split and shift^44^. External fields have previously been studied by a wide range of quantum mechanical phenomena in many areas, including physics, chemistry, biology, material science, engineering, mathematics^45–51^ and others.

Considering the vast applicability of the Manning-Rosen and Schioberg potentials, it is necessary to investigate the bound state solutions of the two-dimensional (2D) SE with the combined potential under the influence of external magnetic and Aharonov–Bohm (AB) fields and their effects on the Shannon entropy and Fisher information for some selected diatomic molecules. The bound state solutions will be obtain using the Nikiforov-Uvarov-Functional Analysis (NUFA) and supersymmetric quantum mechanics (SUYSQM) methods.

This paper is organized as follows: first, we provide detailed solutions to the 2D SE with Manning-Rosen plus Schioberg potential (SPMR) in the presence of magnetic and Aharonov–Bohm (AB) flux fields using the NUFA method. Second, we used the SUYSQM method to obtain the analytical solution of the SE with the combined potential in the presence of magnetic and Aharonov–Bohm (AB) flux fields. Also, the normalized wavefunction obtained is applied to investigate the Shannon entropy and Fisher information in the presence and absence of external magnetic and Aharonov–Bohm (AB) flux fields. Finally, the concluding remarks. The SPMR is of the form.

3 $$V\left( r \right) = D\left[ {1 - \sigma_{0} \left( {\frac{{1 + e^{ - \alpha r} }}{{1 - e^{ - \alpha r} }}} \right)} \right]^{2} - \left( {\frac{{c_{1} e^{ - 2\alpha r} + c_{2} e^{ - \alpha r} }}{{\left( {1 - e^{ - \alpha r} } \right)^{2} }}} \right)$$

## Nikiforov-Uvarov-Functional Analysis (NUFA) method

The Nikiforov-Uvarov Functional Analysis (NUFA) method recently developed by Ikot et al.^52^ has been very helpful in providing solutions for exponential type potentials both in relativistic and nonrelativistic wave equations When using this method to solve either the Schrödinger or Klein–Gordon equation, the energy eigen equation is directly presented in a factorized, closed and compact form. This gives the method an edge over other methods. Meanwhile, the NUFA theory involves solving second order Schrödinger-like differential equation through the analytical combination of Nikiforov-Uvarov (NU) method and functional analysis approach^53–55^. NU is applied to solve a second-order differential equation of the form

4 $$\frac{{d^{2} {\uppsi }\left( {\text{s}} \right)}}{{ds^{2} }} + \frac{{\tilde{\tau }\left( s \right)}}{\sigma \left( s \right)}\frac{{d{\uppsi }\left( {\text{s}} \right)}}{ds} + \frac{{\tilde{\sigma }\left( s \right)}}{{\sigma^{2} \left( s \right)}}\psi \left( s \right) = 0$$

where $\sigma (s)$ and $\widetilde{\sigma }\left(s\right)$ are polynomials at most degree two and $\widetilde{\tau }(s)$ is a first-degree polynomial. Tezean and Sever^56^ latter introduced the parametric form of NU method in the form

5 $$\frac{{d^{2} \psi (s)}}{{ds^{2} }} + \frac{{\alpha_{1} - \alpha_{2} s}}{{s(1 - \alpha_{3} s)}}\frac{{d^{2} \psi (s)}}{{ds^{2} }} + \frac{1}{{s^{2} (1 - \alpha_{3} s)^{2} }}\left[ { - U_{1} s^{2} + U_{2} s - U_{3} } \right]\psi (s) = 0,$$

where $\alpha_{i}$ and $\xi_{i} (i = 1,2,3)$ are all parameters. The differential Eq. (3) has two singularities which is at $s \to 0$ and $s \to \frac{1}{{\alpha_{3} }}$ thus, the wave function can be expressed in the form.

6 $$\Psi_{n} (s) = s^{\lambda } (1 - \alpha_{3} s)^{v} f(s)$$

Substituting Eq. (6) into Eq. (5) and simplifying culminate to the following equation,

7 $$\begin{aligned} s(1 - \alpha_{3} s)\frac{{d^{2} f(s)}}{{ds^{2} }} & + \left[ {\alpha_{1} + 2\lambda - (2\lambda \alpha_{3} + 2v\alpha_{3} + \alpha_{2} )s} \right]\frac{df(s)}{{ds}} \\ & - \alpha_{3} \left( {\lambda + v + \frac{1}{2}\left( {\frac{{\alpha_{2} }}{{\alpha_{3} }} - 1} \right) + \sqrt {\frac{1}{4}\left( {\frac{{\alpha_{2} }}{{\alpha_{3} }} - 1} \right)^{2} + \frac{{U_{1} }}{{\alpha_{3}^{2} }}} } \right)\\&\quad \left( {\lambda + v + \frac{1}{2}\left( {\frac{{\alpha_{2} }}{{\alpha_{3} }} - 1} \right) - \sqrt {\frac{1}{4}\left( {\frac{{\alpha_{2} }}{{\alpha_{3} }} - 1} \right)^{2} + \frac{{U_{1} }}{{\alpha_{3}^{2} }}} } \right)f\left( s \right) \\ & + \left[ {\frac{{\lambda (\lambda - 1) + \alpha_{1} \lambda - U_{3} }}{s} + \frac{{v(v - 1)\alpha_{3} + \alpha_{2} v - \alpha_{1} \alpha_{3} v - \frac{{U_{1} }}{{\alpha_{3} }} + U_{2} - U_{3} \alpha_{3} }}{{\left( {1 - \alpha_{3} s} \right)}}} \right]f\left( s \right) = 0 \\ \end{aligned}$$

Equation (7) can be reduced to a Guassian- hypergeometric equation if and only if the following functions vanished

8 $$\lambda \left( {\lambda - 1} \right) + \alpha_{1} \lambda - U_{3} = 0$$

9 $$\upsilon \left( {\upsilon - 1} \right)\alpha_{3} + \alpha_{2} \upsilon - \alpha_{1} \alpha_{3} \upsilon - \frac{{U_{1} }}{{\alpha_{3} }} + U_{2} - U_{3} \alpha_{3} = 0.$$

Applying the condition of Eq. (8) and Eq. (9) into Eq. (7) results into Eq. (10)

10 $$\begin{aligned} s(1 - \alpha_{3} s) & \frac{{d^{2} f(s)}}{{ds^{2} }}\left[ {\alpha_{1} + 2\lambda - (2\lambda \alpha_{3} + 2v\alpha_{3} + \alpha_{2} )s} \right]\frac{df(s)}{{ds}} \\ & \;\; - \alpha_{3} \left( {\lambda + v + \frac{1}{2}\left( {\frac{{\alpha_{2} }}{{\alpha_{3} }} - 1} \right) + \sqrt {\frac{1}{4}\left( {\frac{{\alpha_{2} }}{{\alpha_{3} }} - 1} \right)^{2} + \frac{{U_{1} }}{{\alpha_{3}^{2} }}} } \right)\\ & \quad\left( {\lambda + v + \frac{1}{2}\left( {\frac{{\alpha_{2} }}{{\alpha_{3} }} - 1} \right) - \sqrt {\frac{1}{4}\left( {\frac{{\alpha_{2} }}{{\alpha_{3} }} - 1} \right)^{2} + \frac{{U_{1} }}{{\alpha_{3}^{2} }}} } \right)f\left( s \right) = 0 \\ \end{aligned}$$

The solutions of Eqs. (8) and (9) are given as

11 $$\lambda = \frac{1}{2}\left( {\left( {1 - \alpha_{1} } \right) \pm \sqrt {\left( {1 - \alpha_{1} } \right)^{2} + 4U_{3} } } \right)$$

12 $$\upsilon = \frac{1}{{2\alpha_{3} }}\left( {\left( {\alpha_{3} + \alpha_{1} \alpha_{3} - \alpha_{2} } \right) \pm \sqrt {\left( {\alpha_{3} + \alpha_{1} \alpha_{3} - \alpha_{2} } \right)^{2} + 4\left( {\frac{{U_{1} }}{{\alpha_{3} }} + \alpha_{3} U_{3} - U_{2} } \right)} } \right)$$

Equation (10) is the hypergeometric equation type of the form

13 $$x\left( {1 - x} \right)\frac{{d^{2} f(s)}}{{ds^{2} }} + \left[ {c - \left( {a + b + 1} \right)x} \right]\frac{df(x)}{{dx}} - \left[ {ab} \right]f(x) = 0$$

where a, b and c are given as follows:

14 $$a = \sqrt {\alpha_{3} } \left( {\lambda + v + \frac{1}{2}\left( {\frac{{\alpha_{2} }}{{\alpha_{3} }} - 1} \right) + \sqrt {\frac{1}{4}\left( {\frac{{\alpha_{2} }}{{\alpha_{3} }} - 1} \right)^{2} + \frac{{U_{1} }}{{U_{3}^{2} }}} } \right)$$

15 $$b = \sqrt {\alpha_{3} } \left( {\lambda + v + \frac{1}{2}\left( {\frac{{\alpha_{2} }}{{\alpha_{3} }} - 1} \right) - \sqrt {\frac{1}{4}\left( {\frac{{\alpha_{2} }}{{\alpha_{3} }} - 1} \right)^{2} + \frac{{U_{1} }}{{\alpha_{3}^{2} }}} } \right)$$

16 $$c = \alpha_{1} + 2\lambda$$

Setting either a or b equal to a negative integer – n, the hypergeometric function f(s) turns to a polynomial of degree n. Hence, the hypergeometric function f(s) approaches finite in the following quantum condition, i.e.,$a = - n$ where $n = 0,1,2,3 \ldots n_{\max }$ or $b = - n$.

Using the above quantum condition,

17 $$\sqrt {\alpha_{3} } \left( {\lambda + \upsilon + \frac{1}{2}\left( {\frac{{\alpha_{2} }}{{\alpha_{3} }} - 1} \right) + \sqrt {\frac{1}{4}\left( {\frac{{\alpha_{2} }}{{\alpha_{3} }} - 1} \right)^{2} + \frac{{U_{1} }}{{\alpha_{3}^{2} }}} } \right) = - n$$

18 $$\lambda + \upsilon + \frac{1}{2}\left( {\frac{{\alpha_{2} }}{{\alpha_{3} }} - 1} \right) + \frac{n}{{\sqrt {\alpha_{3} } }} = - \sqrt {\frac{1}{4}\left( {\frac{{\alpha_{2} }}{{\alpha_{3} }} - 1} \right)^{2} + \frac{{U_{1} }}{{\alpha_{3}^{2} }}}$$

By simplifying Eq. (18), the energy eigen equation using NUFA method is given as

19 $$\lambda^{2} + 2\lambda \left( {\upsilon + \frac{1}{2}\left( {\frac{{\alpha_{2} }}{{\alpha_{3} }} - 1} \right) + \frac{n}{{\sqrt {\alpha_{3} } }}} \right) + \left( {\upsilon + \frac{1}{2}\left( {\frac{{\alpha_{2} }}{{\alpha_{3} }} - 1} \right) + \frac{n}{{\sqrt {\alpha_{3} } }}} \right)^{2} - \frac{1}{4}\left( {\frac{{\alpha_{2} }}{{\alpha_{3} }} - 1} \right)^{2} - \frac{{U_{1} }}{{\alpha_{3}^{2} }} = 0$$

By substituting Eqs. (9) and (10) into Eq. (6), the corresponding wave equation for the NUFA method as

20 $$\Psi_{n} (s) = N_{n} S^{{\frac{{\left( {1 - \alpha_{1} } \right) + \sqrt {\left( {\alpha_{1} - 1} \right)^{2} + 4U_{3} } }}{2}}} \left( {1 - \alpha_{3} } \right)^{{\frac{{\left( {\alpha_{3} + \alpha_{1} \alpha_{3} - \alpha_{2} } \right) + \sqrt {\left( {\alpha_{3} + \alpha_{1} \alpha_{3} - \alpha_{2} } \right)^{2} + 4\left( {\frac{{U_{1} }}{{\alpha_{3}^{2} }} + \alpha_{3} U_{3} - U_{2} } \right)} }}{{2\alpha_{2} }}}} {}_{2}F_{1} (a,b,c;s)$$

### Thermomagnetic energy spectra of 2-dimensional Schrödinger equation under the influence Aharanov-Bohm (AB) flux and external magnetic field using NUFA

The thermomagnetic energy spectra of 2-Dimensional Schrödinger equation under the influenced of AB and Magnetic field with SPMR potential can be obtained from charged particle Hamiltonian operator of the form

21 $$\left\{ {\frac{1}{2\mu }\left( {i\hbar \nabla - \frac{e}{c}\vec{A}} \right)^{2} + D\left[ {1 - \sigma_{0} \left( {\frac{{1 + e^{ - \alpha r} }}{{1 - e^{ - \alpha r} }}} \right)} \right]^{2} - \left( {\frac{{c_{1} e^{ - 2\alpha r} + c_{2} e^{ - \alpha r} }}{{\left( {1 - e^{ - \alpha r} } \right)^{2} }}} \right)} \right\}R\left( {r, \varphi } \right) = E_{nm} R\left( {r, \varphi } \right)$$

$E_{nm}$ is the thermomagnetic energy spectra, $e$ and $\mu$ represent the charge of the particle and the reduced mass respectively. $c$ is the speed of light. Meanwhile, The vector potential $\overrightarrow{A}=\left({A}_{r},{A}_{\phi }, {A}_{z}\right)$ can be written as the superposition of two terms such that $\overrightarrow{A}=\overrightarrow{{A}_{1}}+\overrightarrow{{A}_{2}}$ is the vector potential with azimuthal components such that $\overrightarrow{{A}_{1}}=$ and $\overrightarrow{{A}_{2}}=$, corresponding to the extra magnetic flux $\Phi_{AB}$ generated by a solenoid with $\overrightarrow{\nabla }.\overrightarrow{{A}_{2}}=0$ and $\overrightarrow{B}$ is the magnetic vector field accompanied by $\overrightarrow{\nabla }\times \overrightarrow{{A}_{1}}=\overrightarrow{B}$ ,$\overrightarrow{\nabla }\times \overrightarrow{{A}_{2}}=0$. The vector potential $\overrightarrow{A}$ can then be expressed as

22 $$\vec{A} = \left( {0,\frac{{Be^{ - \alpha r} \hat{\varphi }}}{{1 - e^{ - \alpha r} }} + \frac{{\Phi_{AB} }}{2\pi r}\hat{\varphi },0} \right) = \left( {\frac{{Be^{ - \alpha r} \hat{\varphi }}}{{1 - e^{ - \alpha r} }} + \frac{{\Phi_{AB} }}{2\pi r}\hat{\varphi }} \right)$$

The Laplacian operator and the wave function in cylindrical coordinate is given as

23 $$\begin{aligned} \nabla^{2} & = \frac{{\partial^{2} }}{{\partial r^{2} }} + \frac{1}{r}\frac{\partial }{\partial r} + \frac{1}{{r^{2} }}\frac{{\partial^{2} }}{{\partial \varphi^{2} }} + \frac{{\partial^{2} }}{{\partial z^{2} }} \\ \Psi \left( {r,\varphi } \right) & = \frac{1}{{\sqrt {2\pi r} }}R_{nm} (r)e^{im\varphi } \\ \end{aligned}$$

where $m$ represents the magnetic quantum number. Substituting Eqs. (23) and (22) into Eq. (21) and with much algebraic simplification gives rise to the Schrödinger -like equation of the form

24 $$\frac{{d^{2} R_{nm} (r)}}{{dr^{2} }} + \frac{2\mu }{{h^{2} }}\left[ \begin{gathered} E_{nm} - D\left[ {1 - \sigma_{0} \left( {\frac{{1 + e^{ - \alpha r} }}{{1 - e^{ - \alpha r} }}} \right)} \right]^{2} + \left( {\frac{{c_{1} e^{ - 2\alpha r} + c_{2} e^{ - \alpha r} }}{{\left( {1 - e^{ - \alpha r} } \right)^{2} }}} \right) - \hbar \omega_{c} \left( {m + \xi } \right)\frac{{e^{ - \alpha r} }}{{\left( {1 - e^{ - \alpha r} } \right)r}} \hfill \\ - \left( {\frac{{\mu \omega_{c}^{2} }}{2}} \right)\frac{{e^{ - 2\alpha r} }}{{\left( {1 - e^{ - \alpha r} } \right)^{2} }} - \frac{{\hbar^{2} }}{2\mu }\left( {\frac{{\left( {m + \xi } \right)^{2} - \frac{1}{4}}}{{r^{2} }}} \right) \hfill \\ \end{gathered} \right]R_{nm} (r) = 0.$$

where $\xi = \frac{{\Phi_{AB} }}{{\phi_{0} }}$ is an absolute value containing the flux quantum $\phi_{0} = \frac{hc}{e}$. The cyclotron frequency is represented by $\omega_{c} = \frac{{e\vec{B}}}{\mu c}$. Equation (24) is not exactly solvable due to the presence of centrifugal barrier $\frac{1}{{r^{2} }}$. In order to provide an analytical approximate solution to Eq. (24), we substitute the modified Greene-Aldrich approximation of the form $\frac{1}{{r^{2} }} = \frac{{\alpha^{2} }}{{\left( {1 - e^{ - \alpha r} } \right)^{2} }}$ into Eq. (24) to deal with the centrifugal barrier. Also, using the coordinate transformation $s = e^{ - \alpha r}$ together with the approximation term, Eq. (24) reduced to the hyper-geometric equation of the form

25 $$\frac{{d^{2} R_{nm} (s)}}{{ds^{2} }} + \frac{{\left( {1 - s} \right)}}{{s\left( {1 - s} \right)}}\frac{{dR_{nm} (s)}}{ds} + \frac{1}{{s^{2} \left( {1 - s} \right)^{2} }}\left\{ \begin{gathered} - \left( {\varepsilon^{2} + \chi_{1} + 2\chi_{1} \sigma_{0} + \chi_{1} \sigma_{0}^{2} - \chi_{2} + \chi_{5} } \right)s^{2} \hfill \\ + \left( {2\varepsilon^{2} + 2\chi_{1} - 2\chi_{1} \sigma_{0}^{2} + \chi_{3} - \chi_{4} } \right)s \hfill \\ - \left( {\varepsilon^{2} + \chi_{1} - 2\chi_{1} \sigma_{0} + \chi_{1} \sigma_{0}^{2} + \chi_{6} } \right) \hfill \\ \end{gathered} \right\}R_{nm} (s) = 0.$$

where

26 $$\begin{aligned} \varepsilon^{2} & = - \frac{{2\mu E_{nm} }}{{\hbar^{2} \alpha^{2} }}\begin{array}{*{20}c} , & {\chi_{1} = \frac{2\mu D}{{\hbar^{2} \alpha^{2} }}} \\ \end{array} \begin{array}{*{20}c} , & {\chi_{2} = \frac{{2\mu c_{1} }}{{\hbar^{2} \alpha^{2} }}} \\ \end{array} \begin{array}{*{20}c} , & {\chi_{3} = \frac{{2\mu c_{2} }}{{\hbar^{2} \alpha^{2} }}} \\ \end{array} \\ \chi_{4} & = \frac{{2\mu \omega_{c} \left( {m + \xi } \right)}}{\hbar \alpha }\begin{array}{*{20}c} , & {\chi_{5} = \frac{{\mu^{2} \omega_{c}^{2} }}{{\hbar^{2} \alpha^{2} }}} \\ \end{array} \begin{array}{*{20}c} , & {\chi_{6} = \left( {m + \xi } \right)^{2} - \frac{1}{4}} \\ \end{array} . \\ \end{aligned}$$

Comparing Eq. (25) with NUFA differential equation in Eq. (5), the following polynomial equations can be obtained.

27 $$\begin{gathered} U_{1} = \left( {\varepsilon^{2} + \chi_{1} + 2\chi_{1} \sigma_{0} + \chi_{1} \sigma_{0}^{2} - \chi_{2} + \chi_{5} } \right)\begin{array}{*{20}c} , & {U_{2} = \left( {2\varepsilon^{2} + 2\chi_{1} - 2\chi_{1} \sigma_{0}^{2} + \chi_{3} - \chi_{4} } \right)} \\ \end{array} \hfill \\ U_{3} = \left( {\varepsilon^{2} + \chi_{1} - 2\chi_{1} \sigma_{0} + \chi_{1} \sigma_{0}^{2} + \chi_{6} } \right),\alpha_{1} = \alpha_{2} = \alpha_{3} = 1. \hfill \\ \end{gathered}$$

Using equation NUFA in Eqs. (11), (12), (14), (15) and (16) the following polynomial equations can be obtained

28 $$\lambda = \sqrt {\varepsilon^{2} + \chi_{1} - 2\chi_{1} \sigma_{0} + \chi_{1} \sigma_{0}^{2} + \chi_{6} } ,$$

29 $$\upsilon = \frac{1}{2} + \frac{1}{2}\sqrt {16\chi_{1} \sigma_{0}^{2} - 4\chi_{2} + 4\chi_{5} + 4\chi_{6} - 4\chi_{3} + 4\chi_{4} + 1} ,$$

30 $$a = \left( \begin{gathered} \sqrt {\varepsilon^{2} + \chi_{1} - 2\chi_{1} \sigma_{0} + \chi_{1} \sigma_{0}^{2} + \chi_{6} } + \frac{1}{2} + \frac{1}{2}\sqrt {16\chi_{1} \sigma_{0}^{2} - 4\chi_{2} + 4\chi_{5} + 4\chi_{6} - 4\chi_{3} + 4\chi_{4} + 1} \hfill \\ + \sqrt {\varepsilon^{2} + \chi_{1} + 2\chi_{1} \sigma_{0} + \chi_{1} \sigma_{0}^{2} - \chi_{2} + \chi_{5} } \hfill \\ \end{gathered} \right),$$

31 $$b = \left( \begin{gathered} \sqrt {\varepsilon^{2} + \chi_{1} - 2\chi_{1} \sigma_{0} + \chi_{1} \sigma_{0}^{2} + \chi_{6} } + \frac{1}{2} + \frac{1}{2}\sqrt {16\chi_{1} \sigma_{0}^{2} - 4\chi_{2} + 4\chi_{5} + 4\chi_{6} - 4\chi_{3} + 4\chi_{4} + 1} \hfill \\ - \sqrt {\varepsilon^{2} + \chi_{1} + 2\chi_{1} \sigma_{0} + \chi_{1} \sigma_{0}^{2} - \chi_{2} + \chi_{5} } \hfill \\ \end{gathered} \right),$$

32 $$c = \left( {1 + 2\sqrt {\varepsilon^{2} + \chi_{1} - 2\chi_{1} \sigma_{0} + \chi_{1} \sigma_{0}^{2} + \chi_{6} } } \right).$$

using Eq. (19), the thermo-magnetic energy eigen equation

33 $$\begin{aligned} \varepsilon^{2} & = \frac{1}{4}\left\{ {\frac{{\left( {n + \frac{1}{2} + \frac{1}{2}\sqrt {16\chi_{1} \sigma_{0}^{2} - 4\chi_{2} + 4\chi_{5} + 4\chi_{6} - 4\chi_{3} + 4\chi_{4} + 1} } \right)^{2} + \chi_{2} - \chi_{5} + \chi_{6} - 4\chi_{1} \sigma_{0} }}{{\left( {n + \frac{1}{2} + \frac{1}{2}\sqrt {16\chi_{1} \sigma_{0}^{2} - 4\chi_{2} + 4\chi_{5} + 4\chi_{6} - 4\chi_{3} + 4\chi_{4} + 1} } \right)}}} \right\}^{2} \\ & \;\;\; + 2\chi_{1} \sigma_{0} - \chi_{1} - \chi_{1} \sigma_{0}^{2} - \chi_{6} \\ \end{aligned}$$

Substituting the parameters of Eq. (26) into Eq. (33), the thermomagnetic energy equation become

34 $$\begin{aligned} E_{nm} & = \frac{{h^{2} \alpha^{2} }}{2\mu }\left( {\left( {m + \xi } \right)^{2} - \frac{1}{4}} \right) + D\left( {\sigma_{0} - 1} \right)^{2} \\ & \;\;\; - \frac{{h^{2} \alpha^{2} }}{8\mu }\left\{ {\frac{\begin{gathered} \left[ {n + \frac{1}{2} + \frac{1}{2}\sqrt {\frac{{32\mu D\sigma_{0}^{2} }}{{h^{2} \alpha^{2} }} - \frac{{8\mu c_{1} }}{{h^{2} \alpha^{2} }} - \frac{{8\mu c_{2} }}{{h^{2} \alpha^{2} }} + \frac{{4\mu^{2} \omega_{c}^{2} }}{{h^{2} \alpha^{2} }} + 4\left( {m + \xi } \right)^{2} + \frac{{8\mu \omega_{c} }}{h\alpha }\left( {m + \xi } \right)} } \right]^{2} \hfill \\ + \frac{{2\mu c_{1} }}{{h^{2} \alpha^{2} }} - \frac{{\mu^{2} \omega_{c}^{2} }}{{h^{2} \alpha^{2} }} - \frac{{8\mu D\sigma_{0} }}{{h^{2} \alpha^{2} }} + \left( {m + \xi } \right)^{2} - \frac{1}{4} \hfill \\ \end{gathered} }{{\left[ {n + \frac{1}{2} + \frac{1}{2}\sqrt {\frac{{32\mu D\sigma_{0}^{2} }}{{h^{2} \alpha^{2} }} - \frac{{8\mu c_{1} }}{{h^{2} \alpha^{2} }} - \frac{{8\mu c_{2} }}{{h^{2} \alpha^{2} }} + \frac{{4\mu^{2} \omega_{c}^{2} }}{{h^{2} \alpha^{2} }} + 4\left( {m + \xi } \right)^{2} + \frac{{8\mu \omega_{c} }}{h\alpha }\left( {m + \xi } \right)} } \right]}}} \right\}^{2} \\ \end{aligned}$$

The 2D nonrelativistic energy eigen equation can be obtained with the condition that $\omega_{c} = \xi = 0$, $m = l + \frac{1}{2}$.

Then Eq. (34) become

34a $$\begin{aligned} E_{nm} & = \frac{{h^{2} \alpha^{2} l\left( {l + 1} \right)}}{2\mu } + D\left( {\sigma_{0} - 1} \right)^{2} \\ & \;\;\; - \frac{{h^{2} \alpha^{2} }}{8\mu }\left\{ {\frac{{\left[ {n + \frac{1}{2} + \frac{1}{2}\sqrt {1 + \frac{{32\mu D\sigma_{0}^{2} }}{{h^{2} \alpha^{2} }} - \frac{{8\mu c_{1} }}{{h^{2} \alpha^{2} }} - \frac{{8\mu c_{2} }}{{h^{2} \alpha^{2} }} + 4l\left( {l + 1} \right)} } \right]^{2} + \frac{{2\mu c_{1} }}{{h^{2} \alpha^{2} }} - \frac{{8\mu D\sigma_{0} }}{{h^{2} \alpha^{2} }} + l\left( {l + 1} \right)}}{{\left[ {n + \frac{1}{2} + \frac{1}{2}\sqrt {1 + \frac{{32\mu D\sigma_{0}^{2} }}{{h^{2} \alpha^{2} }} - \frac{{8\mu c_{1} }}{{h^{2} \alpha^{2} }} - \frac{{8\mu c_{2} }}{{h^{2} \alpha^{2} }} + 4l\left( {l + 1} \right)} } \right]}}} \right\}^{2} \\ \end{aligned}$$

### Special cases

#### Schioberg potential

Substituting $c_{1} = c_{2} = 0$.into Eq. (3), then, the potential reduces to Schioberg potential given as

34b $$V\left( r \right) = D\left[ {1 - \sigma_{0} \left( {\frac{{1 + e^{ - \alpha r} }}{{1 - e^{ - \alpha r} }}} \right)} \right]^{2} .$$

Substituting the same condition to Eq. (34) gives the energy-eigen equation for Schioberg potential under the influence of magnetic and AB field as

34c $$\begin{aligned} E_{nm} & = \frac{{h^{2} \alpha^{2} }}{2\mu }\left( {\left( {m + \xi } \right)^{2} - \frac{1}{4}} \right) + D\left( {\sigma_{0} - 1} \right)^{2} \\ & \;\;\; - \frac{{h^{2} \alpha^{2} }}{8\mu }\left\{ {\frac{{\left[ {n + \frac{1}{2} + \frac{1}{2}\sqrt {\frac{{32\mu D\sigma_{0}^{2} }}{{h^{2} \alpha^{2} }} + \frac{{4\mu^{2} \omega_{c}^{2} }}{{h^{2} \alpha^{2} }} + 4\left( {m + \xi } \right)^{2} + \frac{{8\mu \omega_{c} }}{h\alpha }\left( {m + \xi } \right)} } \right]^{2} - \frac{{\mu^{2} \omega_{c}^{2} }}{{h^{2} \alpha^{2} }} - \frac{{8\mu D\sigma_{0} }}{{h^{2} \alpha^{2} }} + \left( {m + \xi } \right)^{2} - \frac{1}{4}}}{{\left[ {n + \frac{1}{2} + \frac{1}{2}\sqrt {\frac{{32\mu D\sigma_{0}^{2} }}{{h^{2} \alpha^{2} }} + \frac{{4\mu^{2} \omega_{c}^{2} }}{{h^{2} \alpha^{2} }} + 4\left( {m + \xi } \right)^{2} + \frac{{8\mu \omega_{c} }}{h\alpha }\left( {m + \xi } \right)} } \right]}}} \right\}^{2} \\ \end{aligned}$$

#### Manning-Rosen potential

Substituting $D = 0$ into Eq. (3), then the potential reduces to Manning-Rosen potential of the form

34d $$V\left( r \right) = - \left( {\frac{{c_{1} e^{ - 2\alpha r} + c_{2} e^{ - \alpha r} }}{{\left( {1 - e^{ - \alpha r} } \right)^{2} }}} \right)$$

Substituting the same condition to Eq. (34) gives the energy eigen equation of Manning-Rosen potential under the influence of magnetic and AB fields as

35 $$\begin{aligned} E_{nm} & = \frac{{h^{2} \alpha^{2} }}{2\mu }\left( {\left( {m + \xi } \right)^{2} - \frac{1}{4}} \right) \\ & \;\;\; - \frac{{h^{2} \alpha^{2} }}{8\mu }\left\{ {\frac{{\left[ {n + \frac{1}{2} + \frac{1}{2}\sqrt { - \frac{{8\mu c_{1} }}{{h^{2} \alpha^{2} }} - \frac{{8\mu c_{2} }}{{h^{2} \alpha^{2} }} + \frac{{4\mu^{2} \omega_{c}^{2} }}{{h^{2} \alpha^{2} }} + 4\left( {m + \xi } \right)^{2} + \frac{{8\mu \omega_{c} }}{h\alpha }\left( {m + \xi } \right)} } \right]^{2} + \frac{{2\mu c_{1} }}{{h^{2} \alpha^{2} }} - \frac{{\mu^{2} \omega_{c}^{2} }}{{h^{2} \alpha^{2} }} + \left( {m + \xi } \right)^{2} - \frac{1}{4}}}{{\left[ {n + \frac{1}{2} + \frac{1}{2}\sqrt { - \frac{{8\mu c_{1} }}{{h^{2} \alpha^{2} }} - \frac{{8\mu c_{2} }}{{h^{2} \alpha^{2} }} + \frac{{4\mu^{2} \omega_{c}^{2} }}{{h^{2} \alpha^{2} }} + 4\left( {m + \xi } \right)^{2} + \frac{{8\mu \omega_{c} }}{h\alpha }\left( {m + \xi } \right)} } \right]}}} \right\}^{2} \\ \end{aligned}$$

Using Eq. (20), the wave function can be presented in a factorized form as

36 $$\Psi_{nm} (s) = N_{n} S^{\beta } \left( {1 - s} \right)^{{\eta + \frac{1}{2}}} F_{1} \left( {a,b,c;s} \right)$$

where

37 $$\begin{gathered} \beta = \sqrt {\frac{2\mu D}{{h^{2} \alpha^{2} }} - \frac{{2\mu E_{nm} }}{{h^{2} \alpha^{2} }} - \frac{{4\mu D\sigma_{0} }}{{h^{2} \alpha^{2} }} + \frac{{2\mu D\sigma_{0}^{2} }}{{h^{2} \alpha^{2} }} + \left( {m + \xi } \right)^{2} - \frac{1}{4}} \hfill \\ \eta = \sqrt {\frac{{8\mu D\sigma_{0}^{2} }}{{h^{2} \alpha^{2} }} - \frac{{2\mu c_{1} }}{{h^{2} \alpha^{2} }} - \frac{{2\mu c_{2} }}{{h^{2} \alpha^{2} }} + \left( {m + \xi + \frac{{\mu \omega_{c} }}{h\alpha }} \right)^{2} } \hfill \\ \end{gathered}$$

Equation (36) can be expressed in terms of Jacobi polynomial as

38 $$\Psi_{nm} (s) = N_{n} S^{\beta } \left( {1 - s} \right)^{{\eta + \frac{1}{2}}} P_{n}^{{\left( {2\beta ,2\eta } \right)}} (1 - 2s)$$

Equation (38) can be normalized using the expression

39 $$\begin{gathered} \int\limits_{0}^{\infty } {\left| {\Psi_{nm} (s)} \right|^{2} } dr = 1 \hfill \\ \Rightarrow N_{nl}^{2} \int\limits_{0}^{\infty } {(e^{ - \alpha r} )^{2\beta } (1 - e^{ - \alpha r} )^{2\eta + 1} \left| {P_{n}^{{\left( {2\beta ,2\eta } \right)}} \left( {1 - 2e^{ - \alpha r} } \right)} \right|^{2} dr} = 1 \hfill \\ \end{gathered}$$

Using Mathematica 10.0 version, the normalized wave function for ground states, first excited state, second excited state and third excited quantum state can be obtained as follows:

40 $$\Psi_{0,m} (r) = \sqrt {\frac{{\alpha \Gamma \left( {2\beta + 2\eta + 2} \right)}}{{\Gamma \left( {2\beta } \right)\Gamma \left( {2 + 2\eta } \right)}}} \left( {e^{ - \alpha r} } \right)^{\beta } \left( {1 - e^{ - \alpha r} } \right)^{{\eta + \frac{1}{2}}}$$

41 $$\Psi_{1,m} (r) = \sqrt {\frac{{2\alpha \beta \Gamma \left( {3 + 2\beta + 2\eta } \right)\Gamma \left( {2\beta + 2\eta + 2} \right)}}{{\Gamma \left( {3 + 2\eta } \right)\Gamma \left( {2\beta } \right)\Gamma \left( {2 + 2\eta } \right)}}} \left( {e^{ - \alpha r} } \right)^{\beta } \left( {1 - e^{ - \alpha r} } \right)^{{\eta + \frac{1}{2}}} P_{1}^{{\left( {2\beta ,2\eta } \right)}} \left( {1 - 2e^{ - \alpha r} } \right)$$

42 $$\Psi_{2,m} (r) = \sqrt {\frac{{4\alpha \beta \Gamma \left( {5 + 2\beta + 2\eta } \right)\Gamma \left( {2\beta + 2\eta + 3} \right)}}{{\Gamma \left( {5 + 2\eta } \right)\Gamma \left( {3 + 2\beta } \right)\Gamma \left( {3 + 2\eta } \right)}}} \left( {e^{ - \alpha r} } \right)^{\beta } \left( {1 - e^{ - \alpha r} } \right)^{{\eta + \frac{1}{2}}} P_{2}^{{\left( {2\beta ,2\eta } \right)}} \left( {1 - 2e^{ - \alpha r} } \right)$$

43 $$\Psi_{3,m} (r) = \sqrt {\frac{{12\alpha \beta \Gamma \left( {7 + 2\beta + 2\eta } \right)\Gamma \left( {2\beta + 2\eta + 4} \right)}}{{\Gamma \left( {7 + 2\eta } \right)\Gamma \left( {4 + 2\beta } \right)\Gamma \left( {4 + 2\eta } \right)}}} \left( {e^{ - \alpha r} } \right)^{\beta } \left( {1 - e^{ - \alpha r} } \right)^{{\eta + \frac{1}{2}}} P_{3}^{{\left( {2\beta ,2\eta } \right)}} \left( {1 - 2e^{ - \alpha r} } \right)$$

## Thermomagnetic energy spectra of 2-dimensional Schrodinger equation under the influence Aharanov-Bohm (AB) flux and external magnetic field using super symmetric quantum mechanics approach

The supersymmetric approach deals with partner Hamiltonian of the form

44 $$H_{ \pm } = \frac{{p^{2} }}{2m} + V\left( x \right).$$

where $p$ is the momentum and $V\left( x \right)$ is the effective potential. The effective potential can be expressed in terms of super potential as

45 $$V_{eff \pm } \left( x \right) = \phi^{2} \left( x \right) \pm \phi^{\prime}\left( x \right)$$

The ground state energy is obtained as

46 $$\phi^{ - 1} \left( x \right) = Ce^{ - N}$$

where $N$ is the normalization constant which for a very simple case can be determined using the expression

47 $$N\left( x \right) = \int\limits_{{x_{0} }}^{x} {\phi \left( r \right)} dr.$$

However, the super potential satisfies the shape invariance condition

48 $$V_{ + } \left( {a_{0} ,x} \right) = V_{ - } \left( {a_{1} ,x} \right) + R\left( {a_{1} } \right)$$

where $a_{1}$ is a new set of parameter determines from the old set $a_{0}$ through the mapping $f:a_{0} \to a_{1} = f\left( {a_{0} } \right)$.

The total supersymmetric energy is defined as

49 $$E_{n} = \sum\limits_{k = 1}^{n} {R(a_{k} )} .$$

While higher order state solutions are obtained through the expression

50 $$\phi_{n}^{ - 1} \left( {a_{0} ,x} \right) = \mathop \Pi \limits_{k = 0}^{n - 1} \left( {\frac{{A^{ + } \left( {a_{k} } \right)}}{{\left( {E_{k} - E_{k} } \right)^{\frac{1}{2}} }}} \right)\phi_{0}^{ - 1} \left( {a_{n} ,x} \right).$$

where $A^{ + } \left( {a_{k} } \right)$ is a raising ladder operator expressed as

51 $$A^{ + } \left( {a_{k} } \right) = - \frac{\partial }{\partial x} + \phi \left( {a_{k} ,x} \right)$$

Also, the Schrodinger equation under super symmetric quantum mechanics approach is arranged in the form

52 $$- \frac{{d^{2} R_{nm} (r)}}{{dr^{2} }} + V_{eff} (\xi ,\omega_{c} ,r)R_{nl} (r) = \tilde{E}_{nm} R_{nm} (r).$$

With the help of approximation to centrifugal term, Eq. (24) can be re- arranged as follows

53 $$\begin{aligned} & - \frac{{d^{2} R_{nm} (r)}}{{dr^{2} }} + \left[ \begin{gathered} \frac{{2\mu D\sigma_{0}^{2} \left( {1 + e^{ - \alpha r} } \right)^{2} }}{{\hbar^{2} \left( {1 - e^{ - \alpha r} } \right)^{2} }} - \frac{{4\mu D\sigma_{0} \left( {1 + e^{ - \alpha r} } \right)}}{{\hbar^{2} \left( {1 - e^{ - \alpha r} } \right)}} - \frac{2\mu }{{\hbar^{2} }}\left( {\frac{{c_{1} e^{ - 2\alpha r} + c_{2} e^{ - \alpha r} }}{{\left( {1 - e^{ - \alpha r} } \right)^{2} }}} \right) \hfill \\ + \frac{{2\mu \alpha \omega_{c} \left( {m + \xi } \right)e^{ - \alpha r} }}{{\hbar \left( {1 - e^{ - \alpha r} } \right)^{2} }} + \left( {\frac{{\mu^{2} \omega_{c}^{2} }}{{\hbar^{2} }}} \right)\frac{{e^{ - 2\alpha r} }}{{\left( {1 - e^{ - \alpha r} } \right)^{2} }} + \left( {\frac{{\alpha^{2} \left( {\left( {m + \xi } \right)^{2} - \frac{1}{4}} \right)}}{{\left( {1 - e^{ - \alpha r} } \right)^{2} }}} \right) \hfill \\ \end{gathered} \right]\\ &\quad R_{nm} (r) = \left( {\frac{{2\mu E_{nm} }}{{\hbar^{2} }} - \frac{2\mu D}{{\hbar^{2} }}} \right)R_{nm} (r). \end{aligned}$$

Equation (53) can then be compared to Eq. (52) such that

54 $$\begin{aligned} V_{eff} (\xi ,\omega_{c} ,r) & = \frac{{2\mu D\sigma_{0}^{2} \left( {1 + e^{ - \alpha r} } \right)^{2} }}{{\hbar^{2} \left( {1 - e^{ - \alpha r} } \right)^{2} }} - \frac{{4\mu D\sigma_{0} \left( {1 + e^{ - \alpha r} } \right)}}{{\hbar^{2} \left( {1 - e^{ - \alpha r} } \right)}} - \frac{2\mu }{{\hbar^{2} }}\left( {\frac{{c_{1} e^{ - 2\alpha r} + c_{2} e^{ - \alpha r} }}{{\left( {1 - e^{ - \alpha r} } \right)^{2} }}} \right) \\ & \;\;\; + \frac{{2\mu \alpha \omega_{c} \left( {m + \xi } \right)e^{ - \alpha r} }}{{\hbar \left( {1 - e^{ - \alpha r} } \right)^{2} }} + \left( {\frac{{\mu^{2} \omega_{c}^{2} }}{{\hbar^{2} }}} \right)\frac{{e^{ - 2\alpha r} }}{{\left( {1 - e^{ - \alpha r} } \right)^{2} }} + \left( {\frac{{\alpha^{2} \left( {\left( {m + \xi } \right)^{2} - \frac{1}{4}} \right)}}{{\left( {1 - e^{ - \alpha r} } \right)^{2} }}} \right) \\ \tilde{E}_{nm} & = \left( {\frac{{2\mu E_{nm} }}{{\hbar^{2} }} - \frac{2\mu D}{{\hbar^{2} }}} \right) \\ \end{aligned}$$

The proposed super potential that is suitable for the effective potential is given as

55 $$\omega (r) = f - \frac{{ge^{ - \alpha r} }}{{\left( {1 - e^{ - \alpha r} } \right)}} \Rightarrow \omega^{\prime}(r) = \frac{{\alpha ge^{ - \alpha r} }}{{\left( {1 - e^{ - \alpha r} } \right)^{2} }}$$

The supersymmetric partner potential can be obtained as follows :

56 $$\begin{gathered} V_{eff}^{ + } (\xi ,\omega_{c} ,r) = \omega^{2} (r) + \omega^{\prime}(r) = f^{2} - \frac{{2fge^{ - \alpha r} }}{{\left( {1 - e^{ - \alpha r} } \right)}} + \frac{{g^{2} e^{ - 2\alpha r} }}{{\left( {1 - e^{ - \alpha r} } \right)^{2} }} + \frac{{\alpha ge^{ - \alpha r} }}{{\left( {1 - e^{ - \alpha r} } \right)^{2} }} \hfill \\ V_{eff}^{ - } (\xi ,\omega_{c} ,r) = \omega^{2} (r) - \omega^{\prime}(r) = f^{2} - \frac{{2fge^{ - \alpha r} }}{{\left( {1 - e^{ - \alpha r} } \right)}} + \frac{{g^{2} e^{ - 2\alpha r} }}{{\left( {1 - e^{ - \alpha r} } \right)^{2} }} - \frac{{\alpha ge^{ - \alpha r} }}{{\left( {1 - e^{ - \alpha r} } \right)^{2} }} \hfill \\ \end{gathered}$$

Equation (56) obeys shape invariance condition

57 $$V_{ + } \left( {r,a_{j} } \right) = V_{ - } \left( {r,a_{j + 1} } \right) + R\left( {a_{j} } \right)$$

The ground state energy can be obtained by solving the associated Riccati equation. Hence, the supersymmetric partner potential of Eq. (56) has a null ground state energy which implies that

58 $$\begin{aligned} V_{ - } \left( r \right) & = V_{eff} \left( {r,\xi ,\omega_{c} } \right) - \overline{E}_{0k} \\ & \Rightarrow f^{2} - \frac{{2fge^{ - \alpha r} }}{{\left( {1 - e^{ - \alpha r} } \right)}} + \frac{{g^{2} e^{ - 2\alpha r} }}{{\left( {1 - e^{ - \alpha r} } \right)^{2} }} - \frac{{\alpha ge^{ - \alpha r} }}{{\left( {1 - e^{ - \alpha r} } \right)^{2} }} \\ & = \frac{{2\mu D\sigma_{0}^{2} \left( {1 + e^{ - \alpha r} } \right)^{2} }}{{\hbar^{2} \left( {1 - e^{ - \alpha r} } \right)^{2} }} - \frac{{4\mu D\sigma_{0} \left( {1 + e^{ - \alpha r} } \right)}}{{\hbar^{2} \left( {1 - e^{ - \alpha r} } \right)}} - \frac{2\mu }{{\hbar^{2} }}\left( {\frac{{c_{1} e^{ - 2\alpha r} + c_{2} e^{ - \alpha r} }}{{\left( {1 - e^{ - \alpha r} } \right)^{2} }}} \right) \\ & \;\;\; + \frac{{2\mu \alpha \omega_{c} \left( {m + \xi } \right)e^{ - \alpha r} }}{{\hbar \left( {1 - e^{ - \alpha r} } \right)^{2} }} + \left( {\frac{{\mu^{2} \omega_{c}^{2} }}{{\hbar^{2} }}} \right)\frac{{e^{ - 2\alpha r} }}{{\left( {1 - e^{ - \alpha r} } \right)^{2} }} + \left( {\frac{{\alpha^{2} \left( {\left( {m + \xi } \right)^{2} - \frac{1}{4}} \right)}}{{\left( {1 - e^{ - \alpha r} } \right)^{2} }}} \right) - \overline{E}_{0k} . \\ \end{aligned}$$

Expanding Eq. (58) in ascending powers of exponent gives rise to three simultaneous equations of the form

59 $$f^{2} + 2fg + g^{2} = \frac{{2\mu D\sigma_{0}^{2} }}{{\hbar^{2} }} + \frac{{4\mu D\sigma_{0} }}{{\hbar^{2} }} - \frac{{2\mu c_{1} }}{{\hbar^{2} }} + \frac{{\mu^{2} \omega_{c}^{2} }}{{\hbar^{2} }} - \overline{E}_{0,k} .$$

60 $$- f^{2} - 2fg - \alpha g^{2} = \frac{{4\mu D\sigma_{0}^{2} }}{{\hbar^{2} }} - \frac{{2\mu c_{2} }}{{\hbar^{2} }} + \frac{{2\mu \alpha \omega_{c} \left( {m + \xi } \right)}}{\hbar } + 2\overline{E}_{0,k}$$

61 $$\begin{aligned} f^{2} & = \frac{{2\mu D\sigma_{0}^{2} }}{{\hbar^{2} }} - \frac{{4\mu D\sigma_{0} }}{{\hbar^{2} }} + \alpha^{2} \left( {\left( {m + \xi } \right)^{2} - \frac{1}{4}} \right) - \overline{E}_{0,k} \\ \Rightarrow \overline{E}_{0,k} & = \frac{{2\mu D\sigma_{0}^{2} }}{{\hbar^{2} }} - \frac{{4\mu D\sigma_{0} }}{{\hbar^{2} }} + \alpha^{2} \left( {\left( {m + \xi } \right)^{2} - \frac{1}{4}} \right) - f^{2} \\ \end{aligned}$$

The ground state energy is calculated using Eq. (61). Substituting Eq. (61) into (60) as well as Eq. (61) into (59) and simplifying gives the following equation

62 $$- 2fg - \alpha g = \frac{{8\mu D\sigma_{0}^{2} }}{{\hbar^{2} }} - \frac{{8\mu D\sigma_{0} }}{{\hbar^{2} }} - \frac{{2\mu c_{2} }}{{\hbar^{2} }} + \frac{{2\mu \alpha \omega_{c} \left( {m + \xi } \right)}}{\hbar } + 2\alpha^{2} \left( {\left( {m + \xi } \right)^{2} - \frac{1}{4}} \right)$$

63 $$2fg + g^{2} = \frac{{8\mu D\sigma_{0} }}{{\hbar^{2} }} - \frac{{2\mu c_{1} }}{{\hbar^{2} }} + \frac{{\mu^{2} \omega_{c}^{2} }}{{\hbar^{2} }} - \alpha^{2} \left( {\left( {m + \xi } \right)^{2} - \frac{1}{4}} \right)$$

Adding Eq. (62) to Eq. (63) and simplifying gives a quadratic equation of the form:

64 $$g^{2} - \alpha g - \left( {\frac{{8\mu D\sigma_{0}^{2} }}{{\hbar^{2} }} + \frac{{\mu^{2} \omega_{c}^{2} }}{{\hbar^{2} }} - \frac{{2\mu c_{1} }}{{\hbar^{2} }} - \frac{{2\mu c_{2} }}{{\hbar^{2} }} + \frac{{2\mu \alpha \omega_{c} \left( {m + \xi } \right)}}{\hbar } + \alpha^{2} \left( {\left( {m + \xi } \right)^{2} - \frac{1}{4}} \right)} \right) = 0$$

The solution to Eq. (64) is

65 $$g = \alpha \left[ {\frac{1}{2} \pm \frac{1}{2}\sqrt {\frac{{32\mu D\sigma_{0}^{2} }}{{\hbar^{2} \alpha^{2} }} + \frac{{4\mu^{2} \omega_{c}^{2} }}{{\hbar^{2} \alpha^{2} }} - \frac{{8\mu c_{1} }}{{\hbar^{2} \alpha^{2} }} - \frac{{8\mu c_{2} }}{{\hbar^{2} \alpha^{2} }} + \frac{{8\mu \omega_{c} \left( {m + \xi } \right)}}{\hbar \alpha } + 4\left( {m + \xi } \right)^{2} } } \right]$$

Using Eq. (63), the constant $f$ can be evaluated as

66 $$f = \frac{1}{2}\left\{ { - g + \frac{{\left[ {\frac{{8\mu D\sigma_{0} }}{{\hbar^{2} }} - \frac{{2\mu c_{1} }}{{\hbar^{2} }} + \frac{{\mu^{2} \omega_{c}^{2} }}{{\hbar^{2} }} - \alpha^{2} \left( {\left( {m + \xi } \right)^{2} - \frac{1}{4}} \right)} \right]}}{g}} \right\}$$

The excited state energy is calculated using shape invariance condition

67 $$V_{ - } \left( {r,\xi ,\omega_{c} ,g} \right) = V_{eff} \left( {r,\xi ,\omega_{c} } \right) - \overline{E}_{0,k} .$$

If $$g = g_{0} \begin{array}{*{20}c} , & {g_{1} = g_{0} + 1\begin{array}{*{20}c} , & {g_{n} = g_{0} + \alpha n} \\ \end{array} } \\ \end{array}$$. Then using Eq. (67), then, the shape invariance condition equation become

68 $$\begin{aligned} V_{ + } \left( {r,\xi ,\omega_{c} ,g_{0} } \right) & = V_{eff} (r,\xi ,\omega_{c} ) - \frac{{2\mu D\sigma_{0}^{2} }}{{\hbar^{2} }} + \frac{{4\mu D\sigma_{0} }}{{\hbar^{2} }} - \alpha^{2} \left( {\left( {m + \xi } \right)^{2} - \frac{1}{4}} \right) \\ & \;\;\; + \frac{1}{4}\left\{ {g_{0} \pm \frac{{\alpha^{2} \left[ {\frac{{2\mu c_{1} }}{{\hbar^{2} \alpha^{2} }} - \frac{{8\mu D\sigma_{0} }}{{\hbar^{2} \alpha^{2} }} - \frac{{\mu^{2} \omega_{c}^{2} }}{{\hbar^{2} \alpha^{2} }} + \left( {m + \xi } \right)^{2} - \frac{1}{4}} \right]}}{{g_{0} }}} \right\}^{2} \\ \end{aligned}$$

69 $$\begin{aligned} V_{ - } \left( {r,\xi ,\omega_{c} ,g_{1} } \right) & = V_{eff} (r,\xi ,\omega_{c} ) - \frac{{2\mu D\sigma_{0}^{2} }}{{\hbar^{2} }} + \frac{{4\mu D\sigma_{0} }}{{\hbar^{2} }} - \alpha^{2} \left( {\left( {m + \xi } \right)^{2} - \frac{1}{4}} \right) \\ & \;\;\; + \frac{1}{4}\left\{ {g_{1} \pm \frac{{\alpha^{2} \left[ {\frac{{2\mu c_{1} }}{{\hbar^{2} \alpha^{2} }} - \frac{{8\mu D\sigma_{0} }}{{\hbar^{2} \alpha^{2} }} - \frac{{\mu^{2} \omega_{c}^{2} }}{{\hbar^{2} \alpha^{2} }} + \left( {m + \xi } \right)^{2} - \frac{1}{4}} \right]}}{{g_{1} }}} \right\}^{2} \\ \end{aligned}$$

We can then construct the supersymmetric partner potentials of the form

71 $$\begin{aligned} R\left( {g_{1} } \right) & = V_{ + } \left( {r,\xi ,\omega_{c} ,g_{0} } \right) - V_{ - } \left( {r,\xi ,\omega_{c} ,g_{1} } \right) \\ & \Rightarrow \frac{1}{4}\left\{ {g_{0} \pm \frac{{\alpha^{2} \left[ {\frac{{2\mu c_{1} }}{{\hbar^{2} \alpha^{2} }} - \frac{{8\mu D\sigma_{0} }}{{\hbar^{2} \alpha^{2} }} - \frac{{\mu^{2} \omega_{c}^{2} }}{{\hbar^{2} \alpha^{2} }} + \left( {m + \xi } \right)^{2} - \frac{1}{4}} \right]}}{{g_{0} }}} \right\}^{2} \\ &\quad- \frac{1}{4}\left\{ {g_{1} \pm \frac{{\alpha^{2} \left[ {\frac{{2\mu c_{1} }}{{\hbar^{2} \alpha^{2} }} - \frac{{8\mu D\sigma_{0} }}{{\hbar^{2} \alpha^{2} }} - \frac{{\mu^{2} \omega_{c}^{2} }}{{\hbar^{2} \alpha^{2} }} + \left( {m + \xi } \right)^{2} - \frac{1}{4}} \right]}}{{g_{1} }}} \right\}^{2} \\ \end{aligned}$$

72 $$\begin{aligned} R\left( {g_{2} } \right) & = V_{ + } \left( {r,\xi ,\omega_{c} ,g_{1} } \right) - V_{ - } \left( {r,\xi ,\omega_{c} ,g_{2} } \right) \\ & \Rightarrow \frac{1}{4}\left\{ {g_{1} \pm \frac{{\alpha^{2} \left[ {\frac{{2\mu c_{1} }}{{\hbar^{2} \alpha^{2} }} - \frac{{8\mu D\sigma_{0} }}{{\hbar^{2} \alpha^{2} }} - \frac{{\mu^{2} \omega_{c}^{2} }}{{\hbar^{2} \alpha^{2} }} + \left( {m + \xi } \right)^{2} - \frac{1}{4}} \right]}}{{g_{1} }}} \right\}^{2} \\ & \quad - \frac{1}{4}\left\{ {g_{2} \pm \frac{{\alpha^{2} \left[ {\frac{{2\mu c_{1} }}{{\hbar^{2} \alpha^{2} }} - \frac{{8\mu D\sigma_{0} }}{{\hbar^{2} \alpha^{2} }} - \frac{{\mu^{2} \omega_{c}^{2} }}{{\hbar^{2} \alpha^{2} }} + \left( {m + \xi } \right)^{2} - \frac{1}{4}} \right]}}{{g_{2} }}} \right\}^{2} \\ \end{aligned}$$

73 $$\begin{aligned} R\left( {g_{3} } \right) & = V_{ + } \left( {r,\xi ,\omega_{c} ,g_{2} } \right) - V_{ - } \left( {r,\xi ,\omega_{c} ,g_{3} } \right) \\ & \Rightarrow \frac{1}{4}\left\{ {g_{2} \pm \frac{{\alpha^{2} \left[ {\frac{{2\mu c_{1} }}{{\hbar^{2} \alpha^{2} }} - \frac{{8\mu D\sigma_{0} }}{{\hbar^{2} \alpha^{2} }} - \frac{{\mu^{2} \omega_{c}^{2} }}{{\hbar^{2} \alpha^{2} }} + \left( {m + \xi } \right)^{2} - \frac{1}{4}} \right]}}{{g_{2} }}} \right\}^{2} \\ &\quad- \frac{1}{4}\left\{ {g_{3} \pm \frac{{\alpha^{2} \left[ {\frac{{2\mu c_{1} }}{{\hbar^{2} \alpha^{2} }} - \frac{{8\mu D\sigma_{0} }}{{\hbar^{2} \alpha^{2} }} - \frac{{\mu^{2} \omega_{c}^{2} }}{{\hbar^{2} \alpha^{2} }} + \left( {m + \xi } \right)^{2} - \frac{1}{4}} \right]}}{{g_{3} }}} \right\}^{2} \\ \end{aligned}$$

74 $$\begin{aligned} R\left( {g_{n} } \right) & = V_{ + } \left( {r,\xi ,\omega_{c} ,g_{n - 1} } \right) - V_{ - } \left( {r,\xi ,\omega_{c} ,g_{n} } \right) \\ & \Rightarrow \frac{1}{4}\left\{ {g_{n - 1} \pm \frac{{\alpha^{2} \left[ {\frac{{2\mu c_{1} }}{{\hbar^{2} \alpha^{2} }} - \frac{{8\mu D\sigma_{0} }}{{\hbar^{2} \alpha^{2} }} - \frac{{\mu^{2} \omega_{c}^{2} }}{{\hbar^{2} \alpha^{2} }} + \left( {m + \xi } \right)^{2} - \frac{1}{4}} \right]}}{{g_{n - 1} }}} \right\}^{2} \\ &\quad- \frac{1}{4}\left\{ {g_{n} \pm \frac{{\alpha^{2} \left[ {\frac{{2\mu c_{1} }}{{\hbar^{2} \alpha^{2} }} - \frac{{8\mu D\sigma_{0} }}{{\hbar^{2} \alpha^{2} }} - \frac{{\mu^{2} \omega_{c}^{2} }}{{\hbar^{2} \alpha^{2} }} + \left( {m + \xi } \right)^{2} - \frac{1}{4}} \right]}}{{g_{n} }}} \right\}^{2} \\ \end{aligned}$$

Recall that $$g = g_{0} \begin{array}{*{20}c} , & {g_{n} = g_{0} + \alpha n} \\ \end{array} = g + \alpha n$$. Using Eq. (74), the higher order supersymmetric energy can be evaluated as

75 $$\overline{E}_{nk} = \frac{1}{4}\left\{ {g_{0} \pm \frac{{\alpha^{2} \left[ {\frac{{2\mu c_{1} }}{{\hbar^{2} \alpha^{2} }} - \frac{{8\mu D\sigma_{0} }}{{\hbar^{2} \alpha^{2} }} - \frac{{\mu^{2} \omega_{c}^{2} }}{{\hbar^{2} \alpha^{2} }} + \left( {m + \xi } \right)^{2} - \frac{1}{4}} \right]}}{{g_{0} }}} \right\}^{2} - \frac{1}{4}\left\{ {g_{n} \pm \frac{{\alpha^{2} \left[ {\frac{{2\mu c_{1} }}{{\hbar^{2} \alpha^{2} }} - \frac{{8\mu D\sigma_{0} }}{{\hbar^{2} \alpha^{2} }} - \frac{{\mu^{2} \omega_{c}^{2} }}{{\hbar^{2} \alpha^{2} }} + \left( {m + \xi } \right)^{2} - \frac{1}{4}} \right]}}{{g_{n} }}} \right\}^{2}$$

Meanwhile, the total energy is the ground state energy plus higher order supesymmetric energy

76 $$\tilde{E}_{nm} = \sum\limits_{k = 1}^{n} {R(a_{k} )} = \overline{E}_{0k} + \overline{E}_{nk}$$

Substituting Eqs. (61) and (75) together with Eq. (66) into Eq. (76) and simplifying gives the total energy as

77 $$\tilde{E}_{nm} = \frac{{2\mu D\sigma_{0}^{2} }}{{\hbar^{2} }} - \frac{{4\mu D\sigma_{0} }}{{\hbar^{2} }} + \alpha^{2} \left( {\left( {m + \xi } \right)^{2} - \frac{1}{4}} \right) - \frac{1}{4}\left\{ {g_{n} \pm \frac{{\alpha^{2} \left[ {\frac{{2\mu c_{1} }}{{\hbar^{2} \alpha^{2} }} - \frac{{8\mu D\sigma_{0} }}{{\hbar^{2} \alpha^{2} }} - \frac{{\mu^{2} \omega_{c}^{2} }}{{\hbar^{2} \alpha^{2} }} + \left( {m + \xi } \right)^{2} - \frac{1}{4}} \right]}}{{g_{n} }}} \right\}^{2}$$

Using the supersymmetric mapping $g_{n} : \to g + \alpha n$ with the total energy expressed as $\tilde{E}_{nm} = \left( {\frac{{2\mu E_{nm} }}{{\hbar^{2} }} - \frac{2\mu D}{{\hbar^{2} }}} \right)$, Eq. (77) now become

78 $$\begin{aligned} \left( {\frac{{2\mu E_{nm} }}{{\hbar^{2} }} - \frac{2\mu D}{{\hbar^{2} }}} \right) & = \frac{{2\mu D\sigma_{0}^{2} }}{{\hbar^{2} }} - \frac{{4\mu D\sigma_{0} }}{{\hbar^{2} }} + \alpha^{2} \left( {\left( {m + \xi } \right)^{2} - \frac{1}{4}} \right) \\ & \;\; - \frac{1}{4}\left\{ {\left( {g + \alpha n} \right) \pm \frac{{\alpha^{2} \left[ {\frac{{2\mu c_{1} }}{{\hbar^{2} \alpha^{2} }} - \frac{{8\mu D\sigma_{0} }}{{\hbar^{2} \alpha^{2} }} - \frac{{\mu^{2} \omega_{c}^{2} }}{{\hbar^{2} \alpha^{2} }} + \left( {m + \xi } \right)^{2} - \frac{1}{4}} \right]}}{{\left( {g + \alpha n} \right)}}} \right\}^{2} \\ \end{aligned}$$

Substituting the value of $g$ from Eq. (65) into Eq. (78) and factorizing gives

79 $$\begin{aligned} E_{nm} & = \frac{{h^{2} \alpha^{2} }}{2\mu }\left( {\left( {m + \xi } \right)^{2} - \frac{1}{4}} \right) + D + D\sigma_{0}^{2} - 2D\sigma_{0} \\ & \;\;\; - \frac{{h^{2} \alpha^{2} }}{8\mu }\left\{ {\frac{\begin{gathered} \left[ {n + \frac{1}{2} \pm \frac{1}{2}\sqrt {\frac{{32\mu D\sigma_{0}^{2} }}{{h^{2} \alpha^{2} }} - \frac{{8\mu c_{1} }}{{h^{2} \alpha^{2} }} - \frac{{8\mu c_{2} }}{{h^{2} \alpha^{2} }} + \frac{{4\mu^{2} \omega_{c}^{2} }}{{h^{2} \alpha^{2} }} + 4\left( {m + \xi } \right)^{2} + \frac{{8\mu \omega_{c} }}{h\alpha }\left( {m + \xi } \right)} } \right]^{2} \hfill \\ + \frac{{2\mu c_{1} }}{{h^{2} \alpha^{2} }} - \frac{{\mu^{2} \omega_{c}^{2} }}{{h^{2} \alpha^{2} }} - \frac{{8\mu D\sigma_{0} }}{{h^{2} \alpha^{2} }} + \left( {m + \xi } \right)^{2} - \frac{1}{4} \hfill \\ \end{gathered} }{{\left[ {n + \frac{1}{2} \pm \frac{1}{2}\sqrt {\frac{{32\mu D\sigma_{0}^{2} }}{{h^{2} \alpha^{2} }} - \frac{{8\mu c_{1} }}{{h^{2} \alpha^{2} }} - \frac{{8\mu c_{2} }}{{h^{2} \alpha^{2} }} + \frac{{4\mu^{2} \omega_{c}^{2} }}{{h^{2} \alpha^{2} }} + 4\left( {m + \xi } \right)^{2} + \frac{{8\mu \omega_{c} }}{h\alpha }\left( {m + \xi } \right)} } \right]}}} \right\} \\ \end{aligned}$$

With a high level of analytical mathematical accuracy, it can be shown that the energy eigen equation obtained through (NUFA) as shown in Eq. (34) reproduces the exact results obtained through SUSYQM as shown in Eq. (79). This further confirms the accuracy of NUFA method in providing bound state solutions to exponential type potentials. Equation (79) can be presented in a more simplified form as:

80 $$E_{nm} = Q_{1} - Q_{2} \left[ {\left( {n + \delta } \right) + \frac{{Q_{3} }}{{\left( {n + \delta } \right)}}} \right]^{2}$$

where

81 $$\begin{aligned} Q_{1} & = \frac{{h^{2} \alpha^{2} }}{2\mu }\left( {\left( {m + \xi } \right)^{2} - \frac{1}{4}} \right) + D + D\sigma_{0}^{2} - 2D\sigma_{0} \\ Q_{2} & = \frac{{h^{2} \alpha^{2} }}{8\mu } \\ Q_{3} & = \frac{{2\mu c_{1} }}{{h^{2} \alpha^{2} }} - \frac{{\mu^{2} \omega_{c}^{2} }}{{h^{2} \alpha^{2} }} - \frac{{8\mu D\sigma_{0} }}{{h^{2} \alpha^{2} }} + \left( {m + \xi } \right)^{2} - \frac{1}{4} \\ \delta & = \left[ {\frac{1}{2} + \frac{1}{2}\sqrt {\frac{{32\mu D\sigma_{0}^{2} }}{{h^{2} \alpha^{2} }} - \frac{{8\mu c_{1} }}{{h^{2} \alpha^{2} }} - \frac{{8\mu c_{2} }}{{h^{2} \alpha^{2} }} + \frac{{4\mu^{2} \omega_{c}^{2} }}{{h^{2} \alpha^{2} }} + 4\left( {m + \xi } \right)^{2} + \frac{{8\mu \omega_{c} }}{h\alpha }\left( {m + \xi } \right)} } \right] \\ \end{aligned}$$

## Thermomagnetic properties

The thermodynamic properties of quantum systems can be obtained from the exact partition function given by

82 $$Z\left( \beta \right) = \sum\limits_{n = 0}^{\lambda } {e^{{ - \beta E_{n} }} }$$

where, $$\lambda$$ is an upper bound of the vibrational quantum number obtained from the numerical solution of $$\frac{{dE_{n} }}{dn} = 0$$$$,$$ given as $$\lambda = - \delta + \sqrt {Q_{3} }$$, $$\beta = \frac{1}{kT}$$ where $$k$$ and T are Boltzmann constant and absolute temperature respectively. In the classical limit, the summation in Eq. (82) can be replaced with an integral:

83 $$Z(\beta ) = \int\limits_{0}^{\lambda } {e^{{ - \beta E_{n} }} dn} .$$

Using Eq. (83), the partition function can be expressed as

84 $$Z(\beta ) = e^{{\beta \left( {2Q_{2} Q_{3} - Q_{1} } \right)}} \int\limits_{0}^{\lambda } {e^{{\beta \left( {Q_{2} \rho^{2} + \frac{{Q_{2} Q_{3}^{2} }}{{\rho^{2} }}} \right)}} d\rho } ,$$

where $$\rho = n + \delta$$.

Using Mathematica 10.0 version, Eq. (84) can be evaluated as

85 $$\frac{{e^{{2\beta Q_{2} Q_{3} - \beta Q_{1} }} \sqrt \pi \left[ {e^{{2\beta Q_{2} Q_{3} }} erf\left( {\sqrt { - \beta Q_{2} } \lambda + \frac{{Q_{3} \sqrt { - \beta Q_{2} } }}{\lambda }} \right) + e^{{ - 2\beta Q_{2} Q_{3} }} erf\left( {\sqrt { - \beta Q_{2} } \lambda - \frac{{Q_{3} \sqrt { - \beta Q_{2} } }}{\lambda }} \right)} \right]}}{{4\sqrt { - \beta Q_{2} } }}.$$

Using Eq. (85), other thermo-magnetic properties can be obtained as follows

- Vibrational internal mean energy. The vibrational internal mean energy^44^ is defined as
  86 $$U\left( {\beta ,B,\Phi_{AB} } \right) = - \frac{\partial }{\partial \beta }InZ\left( {\beta ,B,\Phi_{AB} } \right)$$
- Free energy. The vibrational free energy^44^ is evaluated as
  87 $$F\left( {\beta ,B,\Phi_{AB} } \right) = - \frac{1}{\beta }InZ\left( {\beta ,B,\Phi_{AB} } \right)$$
- The magnetization at finite temperature^44^ is given as
  88 $$M\left( {\beta ,B,\Phi_{AB} } \right) = \frac{1}{\beta }\left( {\frac{1}{{Z\left( {\beta ,B,\Phi_{AB} } \right)}}} \right)\frac{\partial }{\partial B}\left( {\beta ,B,\Phi_{AB} } \right)$$
- Magnetization of a system at zero temperature in a state ($n,m$) is defined by^44^ as
  89 $$M_{nm} \left( {\beta ,\Phi_{AB} } \right) = \frac{{\partial E_{nm} }}{\partial B}$$
- Magnetic susceptibility^44^ at finite temperature is given as
  90 $$\chi_{nm} \left( {\beta ,B,\Phi_{AB} } \right) = \frac{{\partial \left( {\beta ,B,\Phi_{AB} } \right)}}{\partial B}$$
- Persistent current
  91 $$I\left( \beta \right) = - \frac{e}{hc}\frac{{\partial F\left( {\beta ,B,\Phi_{AB} } \right)}}{{\partial \Phi_{AB} }}$$
- The entropy^44^ of the thermo-magnetic system is given as
  92 $$k\ln Z\left( {\beta ,B,\Phi_{AB} } \right) - k\beta \frac{{\partial Z\left( {\beta ,B,\Phi_{AB} } \right)}}{\partial B}$$
- Specific heat capacity^44^of the system is given as
  93 $$C_{s} \left( {\beta ,B,\Phi_{AB} } \right) = k\beta^{2} \frac{{\partial^{2} }}{{\partial \beta^{2} }}\ln Z\left( {\beta ,B,\Phi_{AB} } \right)$$

## Fisher information entropies

In this section, we shall examine the effects of the Aharanov-Bohm (AB) flux and external magnetic field on Fisher information entropy using the proposed potential. Fisher and other quantum information entropies measure the spread of probability distribution for an allowed quantum mechanical state in a D-dimensional space^57–59^. Fisher information has a lot of applications, including the characterizing of complex signals of quantum mechanical systems, derivation of the equation of motion^60^, investigating the behavior of stock market patterns^61^ as well as providing useful information about localization of quantum mechanical particles in a bounded potential well^62^. Fisher entropy expressed in terms of both momentum and position spaces^63,64^ are:

94 $$I\left( \rho \right) = 4\int\limits_{0}^{\infty } {\left| {\nabla \psi \left( {r,\omega_{c} ,\xi } \right)} \right|^{2} } dr$$

95 $$I\left( \gamma \right) = 4\int\limits_{0}^{\infty } {\left| {\nabla \psi \left( {p,\omega_{c} ,\xi } \right)} \right|^{2} } dp$$

For 2-Dimensional Schrodinger wave equation, the Fisher uncertainty product satisfies the inequality^65^

96 $$I\left( \rho \right)I\left( \gamma \right) \ge 16\left( {\left| m \right| + 1} \right)^{2}$$

For a two dimensional problem, the momentum space wave function is expressed as

97 $$\psi_{nm} \left( {p,\phi_{p} ,\omega_{c} ,\xi } \right) = \frac{1}{{\left( {2\pi } \right)^{{{3 \mathord{\left/ {\vphantom {3 2}} \right. \kern-0pt} 2}}} }}\int\limits_{0}^{\infty } {\int\limits_{0}^{2\pi } {\frac{{R_{nm} \left( {r,\omega_{c} ,\xi } \right)}}{\sqrt r }} e^{{i\left[ {m\phi_{r} - pr\cos \left( {\phi_{r} - \phi_{p} } \right)} \right]}} rdrd\phi_{r} }$$

where the solution of the angular part is expressed interms of Bessel function as

98 $$\int\limits_{0}^{2\pi } {e^{{i\left[ {m\phi_{r} - pr\cos \left( {\phi_{r} - \phi_{p} } \right)} \right]}} } d\phi_{r} = \left( { - 1} \right)^{m} 2\pi J_{m} \left( {pr} \right)e^{{im\phi_{p} }}$$

and $J_{\left| m \right|} \left( {pr} \right)$ is the Bessel function of order $m$.

The momentum space wave function is either obtained through a Fourier transform or through expectation value expression. For the purpose of this work, we shall be considering the simplest case where the magnetic quantum spin $m = 0$. Therefore, for momentum space wave function in 2D for $m = 0$, is calculated using expectation value equation of the form

99 $$I\left( \gamma \right) = 4\int\limits_{0}^{\infty } {\left| {\nabla \psi \left( {r,\omega_{c} ,\xi } \right)} \right|^{2} } r^{2} dr$$

### Analytical evaluation of Fisher information entropies for some quantum state

The normalized ground state wave function under the influenced of Aharanov-Bohm flux and external magnetic field is presented in Eq. (39). The gradient of the normalized ground state wave function is given as

100 $$\nabla \psi_{0} \left( {r,\omega_{c} ,\xi } \right) = - \frac{1}{2}\left( {e^{ - \alpha r} } \right)^{\beta } \left( {1 - e^{ - \alpha r} } \right)^{{ - \frac{1}{2} + \eta }} \alpha \left( {2\left( {e^{ - \alpha r} - 1} \right)\beta - 2\eta - 1} \right)\sqrt {\frac{{\alpha \Gamma \left( {2\beta + 2\eta + 2} \right)}}{{\Gamma \left( {2\beta } \right)\Gamma \left( {2 + 2\eta } \right)}}}$$

Substituting Eq. (100) into Eq. (94) gives the fisher information in position space as

101 $$I(p)_{n = 0} = \frac{{\alpha^{2} \left( {1 + 2\eta } \right)\Gamma \left( {1 + 2\beta } \right)\Gamma \left( {2\beta + 2\eta + 2} \right)}}{{\Gamma \left( {2\beta } \right)\Gamma \left( {2 + 2\eta } \right)\Gamma \left( {2\beta + 2\eta + 1} \right)}}$$

Using Eq. (99), Fisher information in momentum space expressed in terms of polygamma function by the help of Mathematica 10.0 version is given as

102 $$I(\gamma )_{n = 0} = \frac{2}{{\alpha^{2} \left( {1 + 2\eta } \right)}}\left\{ \begin{gathered} \frac{4 + 8\eta }{{\left( {1 + 2\beta + 2\eta } \right)^{2} }} - \left( {\frac{{4\left( {1 + 2\eta } \right)\psi^{\left( 0 \right)} \left( {0,2\beta } \right)}}{{\left( {1 + 2\beta + 2\eta } \right)}}} \right) + \left( {\frac{{\left( {4 + 8\eta } \right)\psi^{\left( 0 \right)} \left( {0,1 + 2\beta + 2\eta } \right)}}{{\left( {1 + 2\beta + 2\eta } \right)}}} \right) \hfill \\ + \left( {2 + 4\eta } \right)\psi^{\left( 0 \right)} \left( {0,2\beta } \right) - 4\left( {1 + 2\eta } \right)\psi^{\left( 0 \right)} \left( {0,2\beta } \right)\psi^{\left( 0 \right)} \left( {0,1 + 2\beta + 2\eta } \right) \hfill \\ + 2\left( {1 + 2\eta } \right)\psi^{\left( 0 \right)} \left( {0,1 + 2\beta + 2\eta } \right)^{2} \hfill \\ + \left( {2 + 4\eta } \right)\psi^{\left( 0 \right)} \left( {1,2\beta } \right) - 2\left( {1 + 2\eta } \right)\psi^{\left( 0 \right)} \left( {1,1 + 2\beta + 2\eta } \right) \hfill \\ \end{gathered} \right\}$$

Using the same procedure, we can obtain Fisher information for other quantum states. For $n = 1$, the Fisher information for both position and momentum spaces are given as

103 $$I(p)_{n = 1} = \frac{{ - 2\alpha^{2} \beta \left( {1 + 2\eta } \right)\Gamma \left( {3 + 2\beta + 2\eta } \right)\Gamma \left( {1 + 6\eta } \right)\Gamma \left( {2\eta } \right)}}{{\Gamma \left( {3 + 2\eta } \right)\Gamma \left( {2 + 2\eta } \right)}}$$

104 $$I(\gamma )_{n = 1} = 4\left[ {\frac{{\Xi_{0} + \Xi_{1} + \Xi_{2} + \Xi_{3} + \Xi_{4} + \Xi_{5} + \Xi_{6} + \Xi_{7} }}{{\alpha^{2} \left( {1 + 2\beta } \right)\left( {1 + 2\eta } \right)\left( {3 + 2\eta } \right)\left( {3 + 2\beta + 2\eta } \right)^{2} }}} \right]$$

where

105 $$\begin{aligned} \Xi_{0} & = 86 + 94\beta - 64\beta^{2} + 42\left( {47 + 56\beta } \right)\eta + 4\left( {43 + 30\beta } \right)\eta^{2} + 16\left( {5 + \beta } \right)\eta^{3} + 16\eta^{4} \\ \Xi_{1} & = \frac{{\beta \left( {1 + 2\beta } \right)^{2} }}{{\left( {1 + \beta + \eta } \right)^{2} }} + \frac{{\left( {1 + 2\beta } \right)\left( {2\beta - 1} \right)\left( {1 + 3\beta } \right)}}{{\left( {1 + \beta + \eta } \right)}} + \frac{{16\left( {\beta - 1} \right)\beta \left( {1 + 2\beta } \right)}}{{\left( {1 + 2\beta + 2\eta } \right)^{2} }} + \frac{{8 + 8\beta \left( {14\beta^{2} - 5} \right)}}{{\left( {1 + 2\beta + 2\eta } \right)}} \\ \Xi_{2} & = \left( {1 + 2\beta } \right)\left( {3 + 2\beta + 2\eta } \right)^{2} \left( {3 + 8\eta + 4\eta^{2} } \right)\psi^{\left( 0 \right)} \left( {0,2\beta } \right)^{2} \\ \Xi_{3} & = \left\{ {\frac{{2\left( {1 + 2\eta } \right)\left( {3 + 2\beta + 2\eta } \right)\left[ \begin{gathered} \beta^{2} \left( {44 + 40\eta } \right) + \beta \left( {3 + 2\eta } \right)\left( {29 + 56\eta + 28\eta^{2} } \right) \hfill \\ + \left( {1 + \eta } \right)2\left( {3 + 2\eta } \right)\left( {7 + 12\eta + 4\eta^{2} } \right) + 4\beta^{2} \left( {28 + 49\eta + 22\eta^{2} } \right)\psi^{\left( 0 \right)} \left( {0,1 + 2\beta + 2\eta } \right) \hfill \\ \end{gathered} \right]}}{{\left( {1 + \beta + \eta } \right)\left( {1 + 2\beta + 2\eta } \right)}}} \right\} \\ \Xi_{4} & = \left( {1 + 2\beta } \right)\left( {3 + 2\beta + 2\eta } \right)^{2} \left( {3 + 8\eta + 4\eta^{2} } \right)\psi^{\left( 0 \right)} \left( {0,1 + 2\beta + 2\eta } \right)^{2} \\ \Xi_{5} & = \left\{ \begin{gathered} \left( {1 + \beta + \eta } \right)\left( {1 + 2\beta + 2\eta } \right)\left( {2 + 4\eta } \right)\left( {3 + 2\beta + 2\eta } \right)\psi^{\left( 0 \right)} \left( {0,2\beta } \right)\beta^{2} \left( {44 + 40\eta } \right) \hfill \\ + \beta \left( {3 + 2\eta } \right)\left( {29 + 56\eta + \eta^{2} } \right) \hfill \\ \end{gathered} \right\}^{ - 1} \\ \Xi_{6} & = \left( {1 + \eta } \right)\left( {3 + 2\eta } \right)\left( {7 + 12\eta + 4\eta^{2} } \right) + 4\beta^{2} \left( {28 + 49\eta + 22\eta^{2} } \right) \\ & \;\;\; + \left( {1 + 2\beta } \right)\left( {1 + \beta + \eta } \right)\left( {3 + 2\eta } \right)\left( {1 + 2\beta + 2\eta } \right)\left( {3 + 2\beta + 2\eta } \right)\psi^{\left( 0 \right)} \left( {0,1 + 2\beta + 2\eta } \right) \\ \Xi_{7} & = \left( {1 + 2\beta } \right)\left( {3 + 2\beta + 2\eta } \right)^{2} \left( {3 + 8\eta + 4\eta^{2} } \right)\psi^{\left( 0 \right)} \left( {1,2\beta } \right) \\ & \;\;\; + \left( {2\beta - 1} \right)\left( {3 + 2\beta + 2\eta } \right)^{2} \left( {3 + 8\eta + 4\eta^{2} } \right)\psi^{\left( 0 \right)} \left( {1,1 + 2\beta + 2\eta } \right) \\ \end{aligned}$$

where the polygamma function is generally expressed as

106 $$\psi^{\left( 0 \right)} (x) = \left( {\ln \Gamma \left( x \right)} \right)^{\prime } = \frac{{\int\limits_{0}^{\infty } {t^{x - 1} } e^{ - t} \ln tdt}}{{\int\limits_{0}^{\infty } {t^{x - 1} } e^{ - t} dt}}$$

## Results and discussion

**Figure 1:**
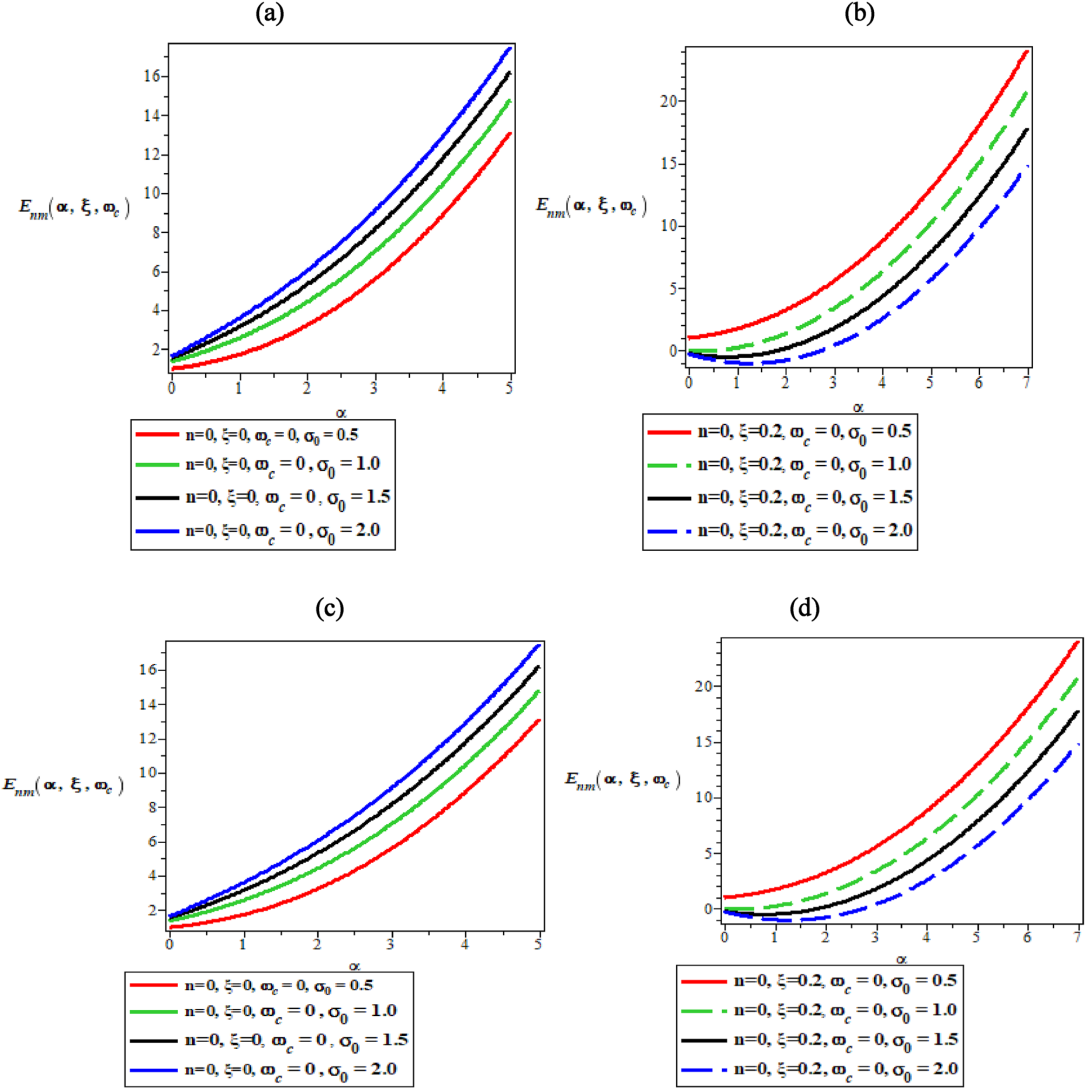
Variation of thermomagnetic energy spectra against the screening parameter in (**a**) the absence of AB and Magnetic field, (**b**) the presence of only magnetic field; (**c**) the presence of only AB field and (**d**) the presence of both magnetic and AB field.

Figures 1a–d are the plots of variation of thermomagnetic energy spectra against the screening parameter in the absence of AB and magnetic field, the presence of the only magnetic field, the presence of only AB field and the presence of both magnetic and AB fields, respectively. In Fig. 1a–d, the bound state energy spectral diagrams all increases monotonically with increasing values of the screening parameter ($\alpha$) in such a unique and quantized manner.

**Figure 2:**
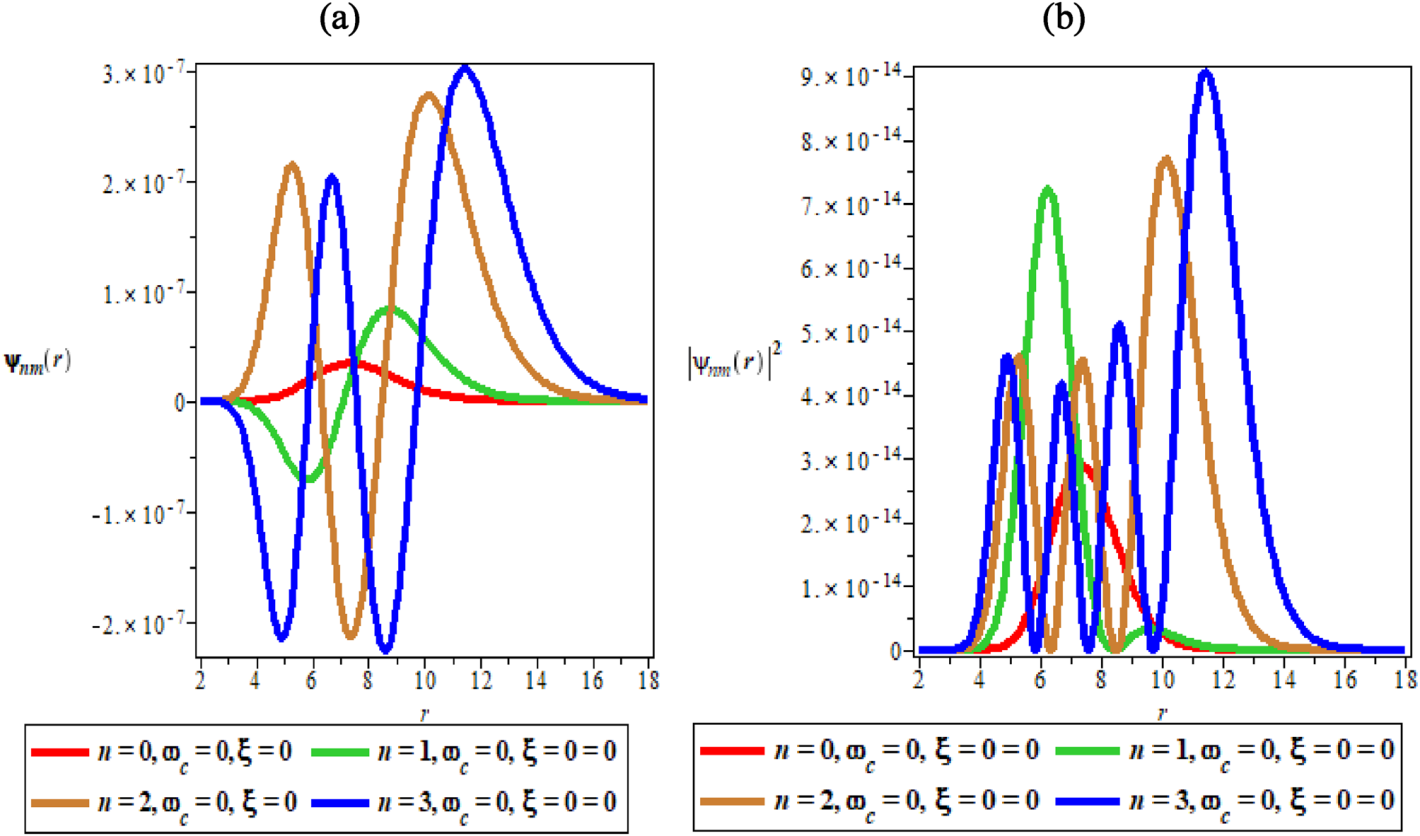
(**a**) The variation of wave function plot against the radial distance in the absence of both AB and magnetic field. (**b**) The variation of probability density plot against the radial distance in absence of both AB and magnetic field.

Figure 2a, b are the variation of wave function plot against the radial distance in the absence of both AB and magnetic field and the variation of probability density plot against the radial distance in absence of both AB and magnetic field, respectively. In Fig. 2a, the wave function showcases intertwining multiple sinusoidal curves representing the different quantum states. In Fig. 2b, the probability density plots in the absence of AB and magnetic field show a normal distribution curve with multiple peaks, each depicting a different quantum state. It is interesting to note that in Fig. 2a, the ground state has the lowest peak, while the highest state ($n = 3$) has the highest peak. Figure 2b agreed excellently with the theoretical and experimental descriptions of probability density. It is expected that in an ideal condition, the peak of the probability density plot should increase as the quantum state increases. This is only possible because in Fig. 2a and b, the wave function and probability density plots are carried out in the absence of AB and magnetic field respectively.

**Figure 3:**
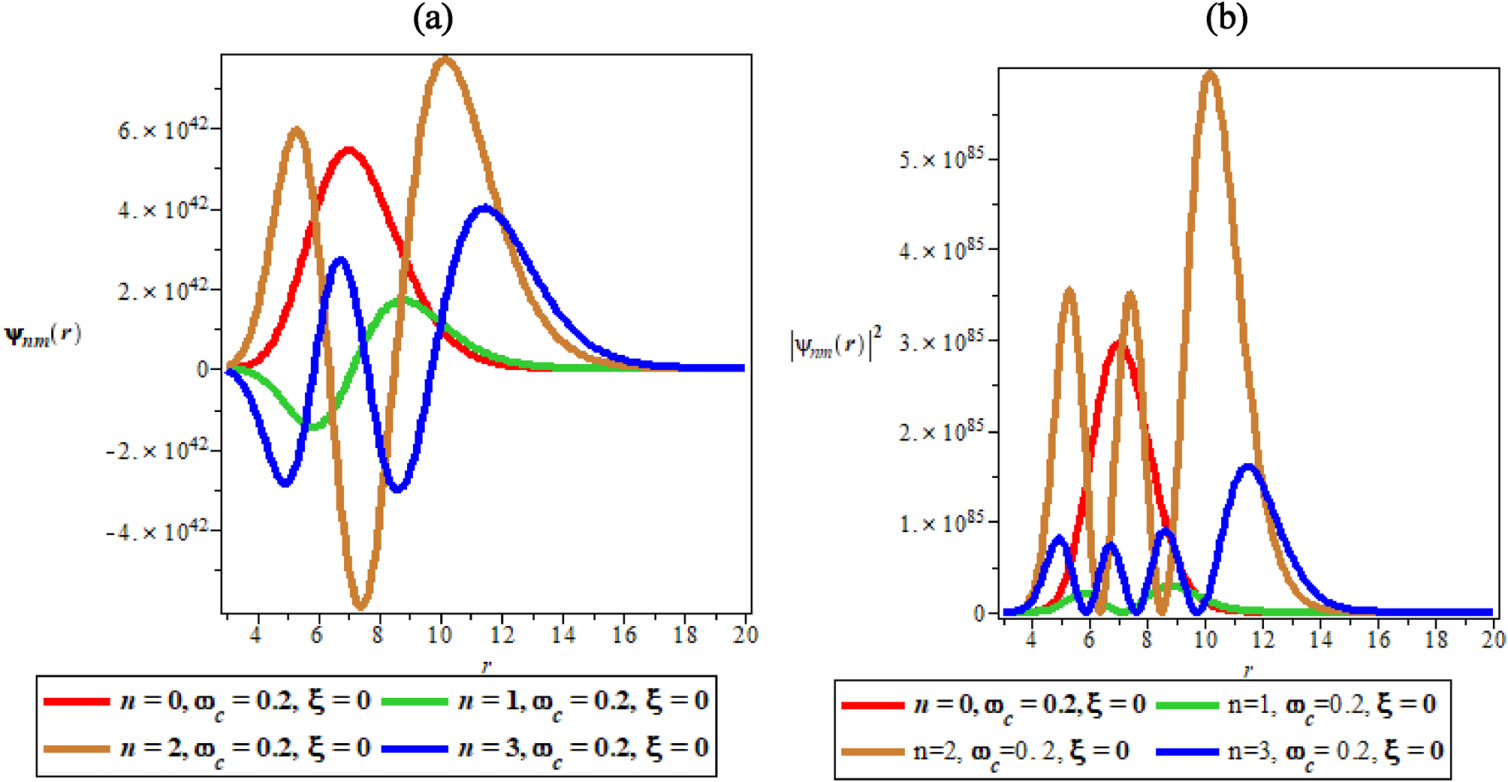
(**a**) The variation of wave function plot against the radial distance in the presence of magnetic field. (**b**) The variation of probability density plot against the radial distance in the presence of magnetic field.

Figure 3a, b are the variations of the wave function and the probability density plots against the radial distance in the presence of magnetic field. Figure 3a shows a periodic and sinusoidal wave function similar to Fig. 2a. However, in Fig. 3b, there is distortion in the probability distribution curves because of the presence of magnetic field. The presence of the magnetic field does not allow uniform distribution of probability density plots in increasing order of the quantum state whose highest peak supposed to occur at ($n = 3$). However, in Fig. 3b, $n = 2$ has the highest peak, followed by $n = 0$ before $n = 3$. The disorderliness, ambiguity and distortions in the peaks clearly show the effect of magnetic field.

**Figure 4:**
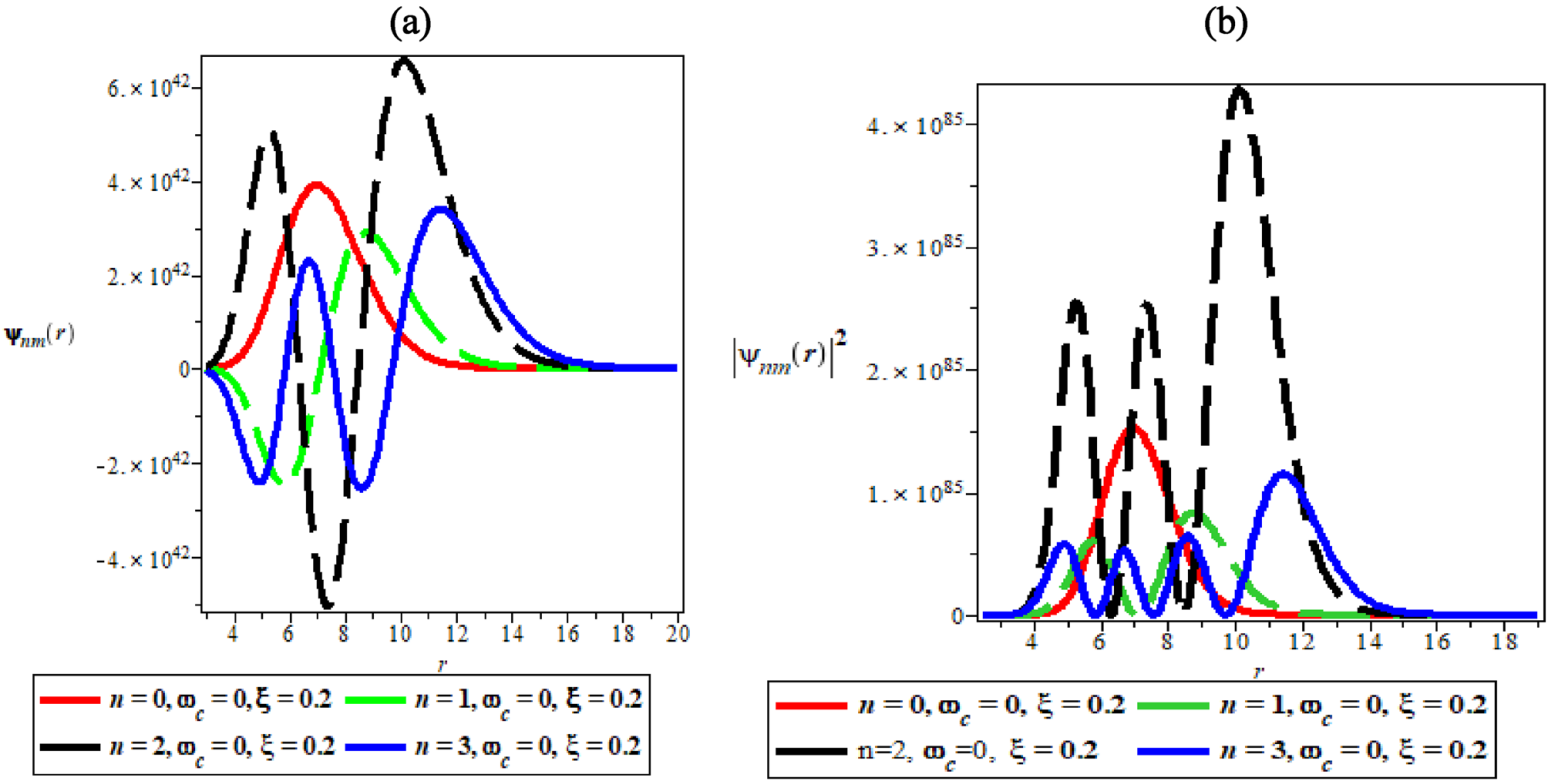
(**a**) The variation of wave function plot against the radial distance in the presence of AB field. (**b**) The variation of probability density plot against the radial distance in the presence of AB field.

Figure 4a, b are the variation of the wave function and probability density plots against the radial distance in the presence of AB field, respectively. Figure 4a and b has similar explanation to Fig. 3a and b when the distortions to the probability density plot are affected by the presence of Aharonov-Bohm flux field.

**Figure 5:**
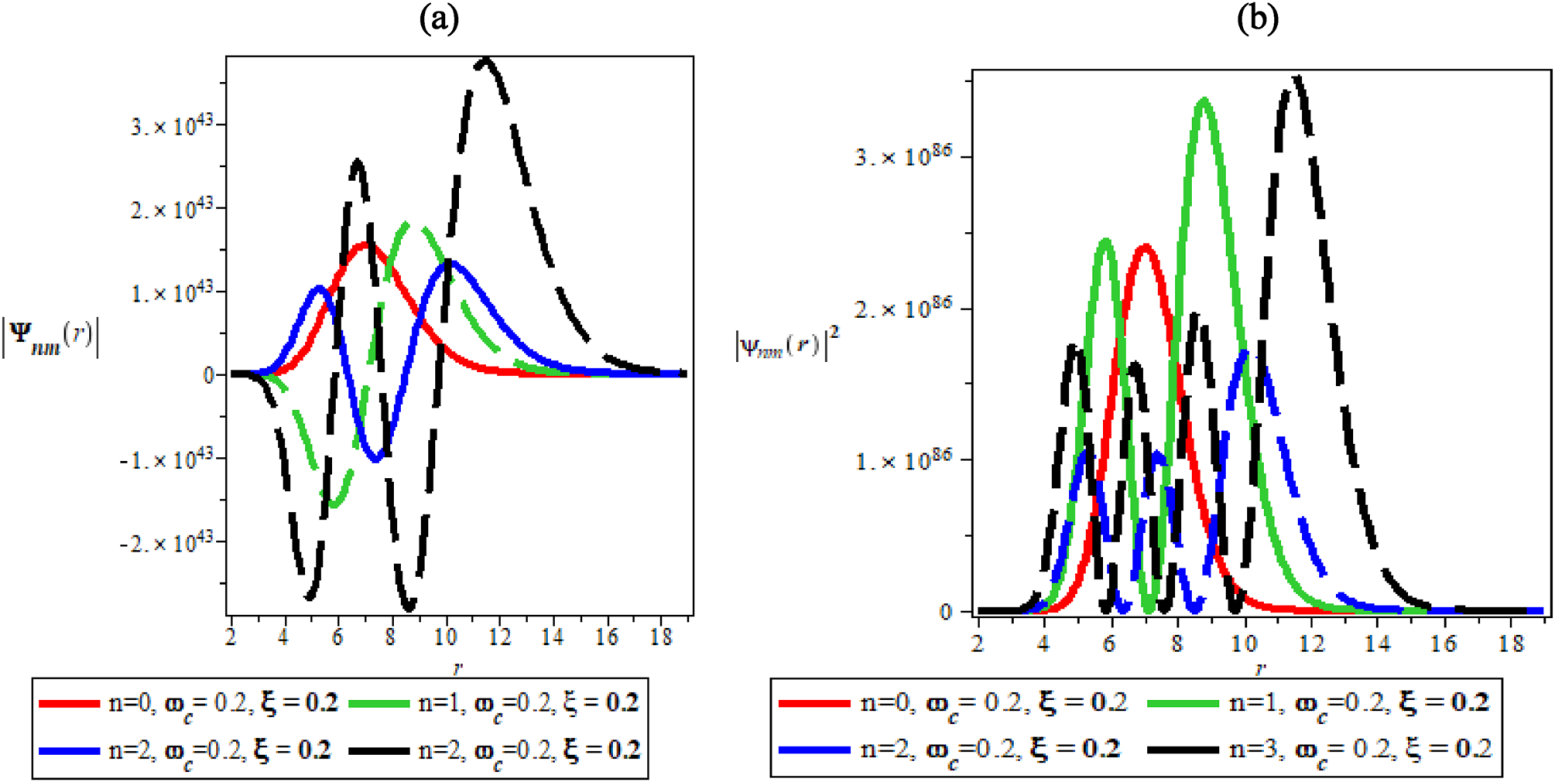
(**a**) The variation of wave function plot against the radial distance in the presence of both magnetic and AB field. (**b**) The variation of probability density plot against the radial distance in the presence of both magnetic and AB field.

Figure 5a, b show how the wave function and probability density varied with radial distance in the presence of both magnetic and AB fields . Under the influence of AB and magnetic fields, the wave function in Fig. 5a is sinusoidal and periodic. .However, in Fig. 5b, something fascinating occurs. The peaks of probability density plot for quantum state ($n = 1$) are almost the same as $n = 2$, i.e., the combined effect of AB and magnetic effect establish quantum state equivalence.

**Figure 6:**
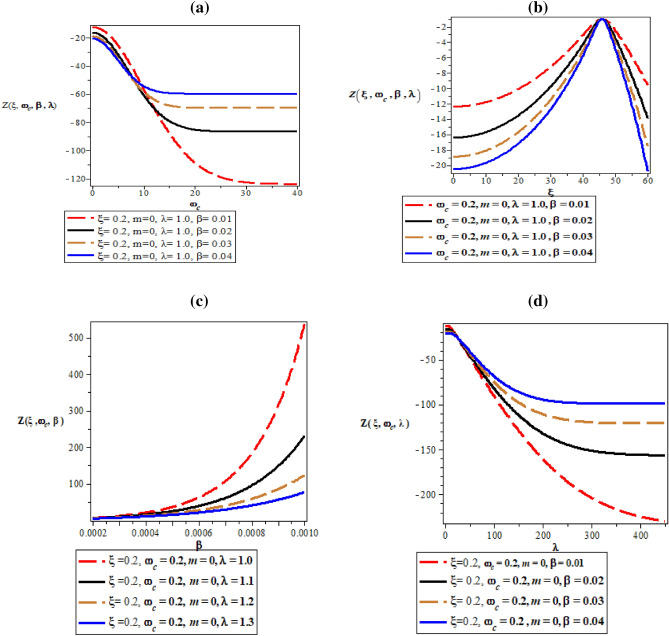
(**a**) Plot of partition function against magnetic flux $\left( {\omega_{c} } \right)$ for different values of inverse temperature parameter $(\beta )$. (**b**) Plot of partition function against AB flux $\left( \xi \right)$ for different values of inverse temperature parameter ($\beta$). (**c**) Plot of partition function against inverse temperature parameter ($\beta$) for fixed value of $\omega_{c}$ and $\xi$ but for different values of maximum vibrational quantum number ($\lambda$). (**d**) Plot of partition function against the maximum vibrational quantum number ($\lambda$) for fixed value of $\omega_{c}$ and $\xi$ but for different values inverse temperature parameter ($\beta$).

Figure 6a–d are the plot of partition function against magnetic flux $\left( {\omega_{c} } \right)$ for different values of inverse temperature parameter $(\beta )$, plot of partition function against AB flux $\left( \xi \right)$ for different values of inverse temperature parameter ($\beta$), plot of partition function against inverse temperature parameter ($\beta$) for fixed value of $\omega_{c}$ and $\xi$ but for different values of maximum vibrational quantum number ($\lambda$) and plot of partition function against the maximum vibrational quantum number ($\lambda$)for fixed value of $\omega_{c}$ and $\xi$ but for different values of inverse temperature parameter ($\beta$), respectively. In Fig. 6a, the partition function starts from the negative y-axis an increase exponentially with increasing value of the magnetic field. The same explanation occurs in Fig. 6d where the partition function increases exponentially with an increase in maximum vibrational quantum number.

In Fig. 6b, the partition function rises monotonically with unique spacing before reaching a peak value with local maximum turning point at $\xi = 40$. In Fig. 6c, the partition function increases monotonically with an increase in inverse temperature parameter.

**Figure 7:**
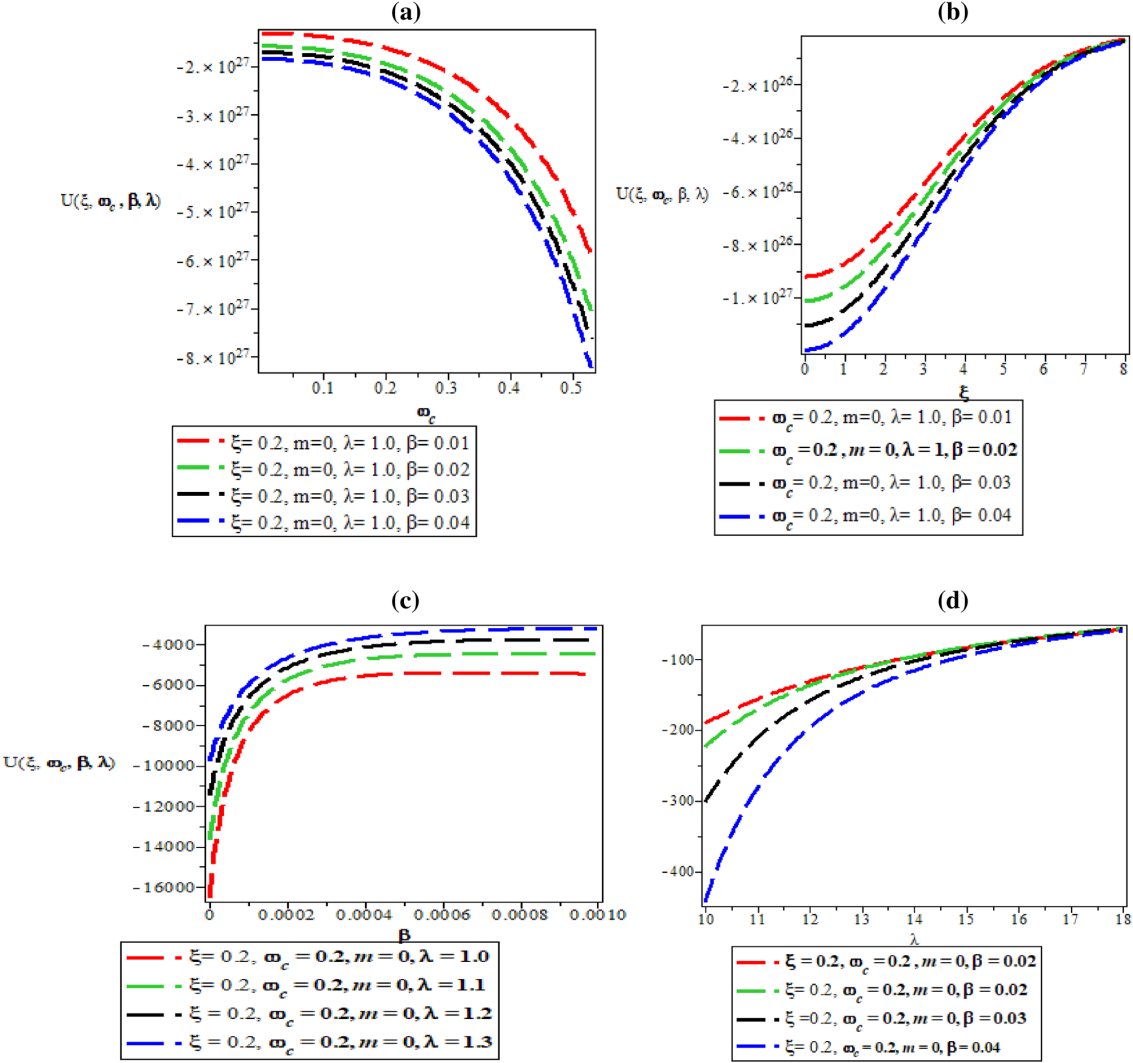
(**a**) Plot of vibrational mean energy against magnetic flux $\left( {\omega_{c} } \right)$ for different values of inverse temperature parameter $(\beta )$. (**b**) Plot of vibrational mean energy against AB flux $\left( \xi \right)$ for different values of inverse temperature parameter ($\beta$). (**c**) Plot of vibrational mean energy against inverse temperature parameter ($\beta$) for fixed value of $\omega_{c}$ and $\xi$ but for different values of maximum vibrational quantum number ($\lambda$). (**d**) Plot of vibrational mean energy against the maximum vibrational quantum number ($\lambda$) for fixed value of $\omega_{c}$ and $\xi$ but for different values inverse temperature parameter ($\beta$).

Figure 7a–d are the plot of vibrational mean energy against magnetic flux $\left( {\omega_{c} } \right)$ for different values of inverse temperature parameter $(\beta )$, plot of vibrational mean energy against AB flux $\left( \xi \right)$ for different values of inverse temperature parameter ($\beta$), plot of vibrational mean energy against inverse temperature parameter ($\beta$) for fixed value of $\omega_{c}$ and $\xi$ but for different values of maximum vibrational quantum number ($\lambda$) and plot of vibrational mean energy against the maximum vibrational quantum number ($\lambda$) for fixed value of $\omega_{c}$ and $\xi$ but for different values inverse temperature parameter ($\beta$) respectively.

In Fig. 7a, the vibrational mean energy showcase a parabolic curve which increases with an increase in magnetic field. In Fig. 7b, the vibrational mean energy increases monotonically before converging at $\xi = 6$ with increase in AB flux. Also, the vibrational mean energy increases uniquely from the origin with quantized spacing, in an increasing value of inverse temperature parameter as shown in Fig. 7c. Correspondingly, the vibrational mean energy also increase with increasing value of maximum vibrational quantum number as presented in Fig. 7d.

**Figure 8:**
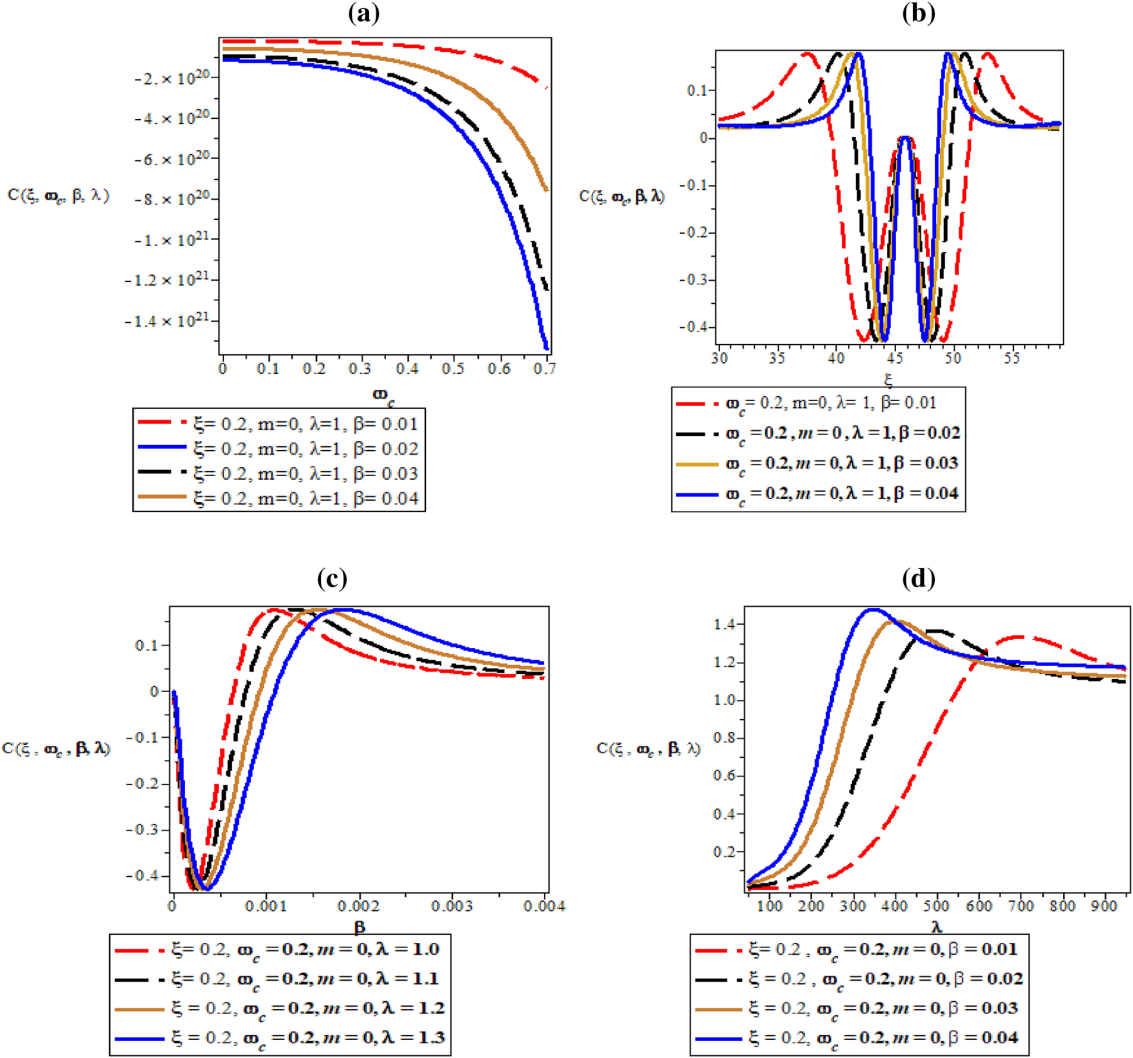
(**a**) Plot of vibrational heat capacity against magnetic flux $\left( {\omega_{c} } \right)$ for different values of inverse temperature parameter $(\beta )$. (**b**) Plot of vibrational heat capacity against AB flux $\left( \xi \right)$ for different values of inverse temperature parameter ($\beta$). (**c**) Plot of vibrational heat capacity against inverse temperature parameter ($\beta$) for fixed value of $\omega_{c}$ and $\xi$ but for different values of maximum vibrational quantum number ($\lambda$). (**d**) Plot of vibrational heat capacity against the maximum vibrational quantum number ($\lambda$) for fixed value of $\omega_{c}$ and $\xi$ but for different values inverse temperature parameter ($\beta$).

Figure 8a–d are the plot of vibrational heat capacity against magnetic flux $\left( {\omega_{c} } \right)$ for different values of inverse temperature parameter $(\beta )$, plot of vibrational heat capacity against AB flux $\left( \xi \right)$ for different values of inverse temperature parameter ($\beta$), plot of vibrational heat capacity against inverse temperature parameter ($\beta$) for fixed value of $\omega_{c}$ and $\xi$ but for different values of maximum vibrational quantum number ($\lambda$) and plot of vibrational heat capacity against the maximum vibrational quantum number ($\lambda$) for fixed value of $\omega_{c}$ and $\xi$ but for different values inverse temperature parameter ($\beta$) respectively.

In Fig. 8a, the vibrational heat capacity increases monotonically with increase in magnetic field. In Fig. 8b, the vibrational heat capacity shows symmetrical curves with common converged maximum point at $\xi = 45$ . This maximum point divides the curves into equal half both in a decreasing and increasing value of $\xi$. The physical interpretation of Fig. 8b is that heat capacity from the concept of molecular vibration relates to the kinetic energy of the molecules of the system. So, the Fig. 8b completely shows that with the influence of Aharanov-Bohm flux field, the kinetic energy of the molecules of the system remains constant during molecular vibrations. This explains why there is a symmetrical curves both at the left and right hand side of the thermomagnetic plot. In Fig. 8c, the vibrational heat capacity is a parabolic curve that concaves upward with minimum turning point at $\beta = 0.0005\;\;{\text{K}}^{ - 1}$ before rising to various local maximum turning points in increasing value of $\beta$, before converging at $\beta = 0.004\;\;{\text{K}}^{ - 1}$. In Fig. 8d, the specific heat capacity increases asymmetrically to various unique maximum point before converging at $\lambda = 1000$ with increasing value of maximum vibrational quantum number.

**Figure 9:**
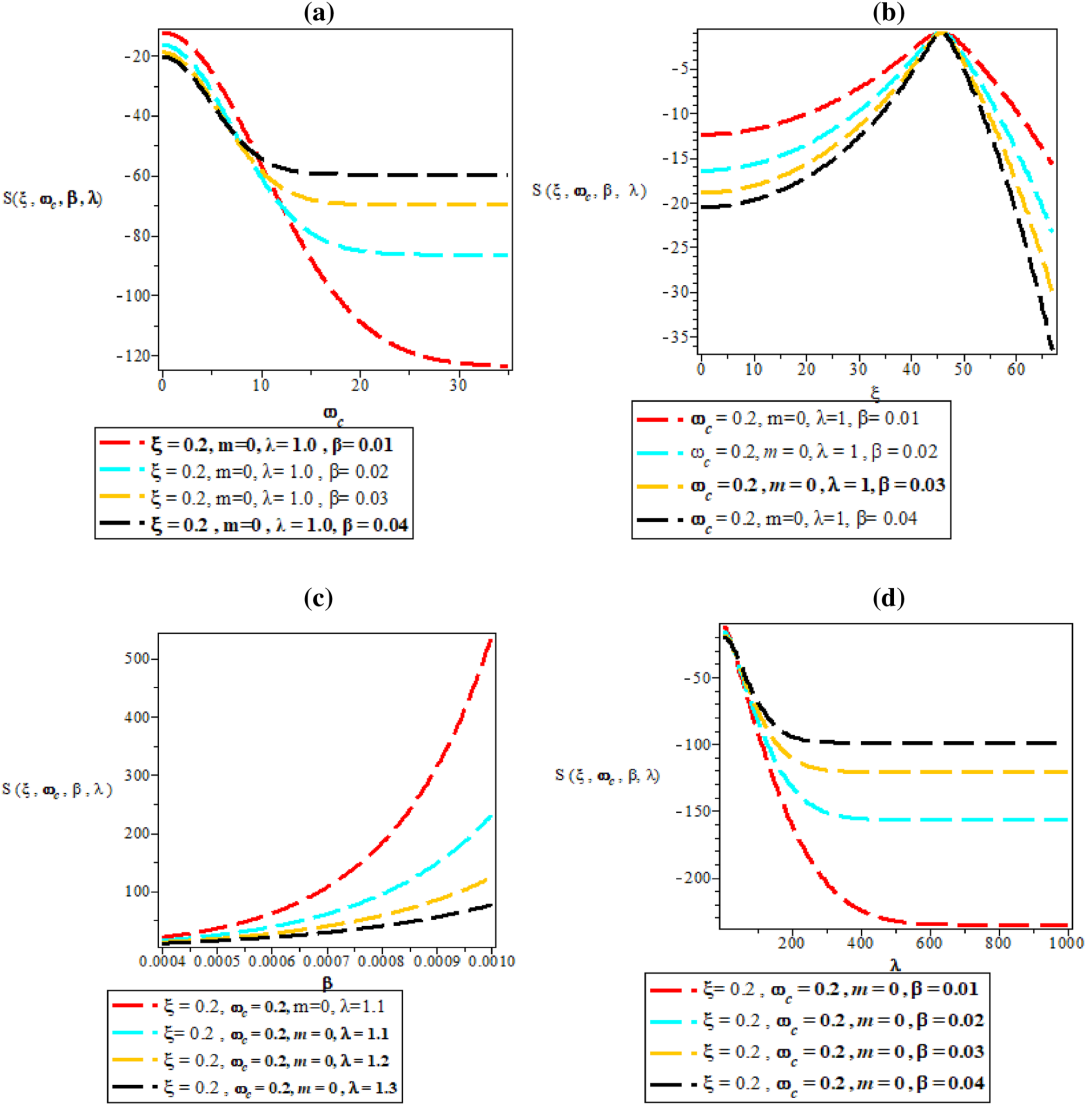
(**a**) Plot of vibrational entropy against magnetic flux $\left( {\omega_{c} } \right)$ for different values of inverse temperature parameter $(\beta )$. (**b**) Plot of vibrational entropy against AB flux $\left( \xi \right)$ for different values of inverse temperature parameter ($\beta$). (**c**) Plot of vibrational entropy against inverse temperature parameter ($\beta$) for fixed value of $\omega_{c}$ and $\xi$ but for different values of maximum vibrational quantum number ($\lambda$). (**d**) Plot of vibrational entropy against the maximum vibrational quantum number ($\lambda$), for fixed value of $\omega_{c}$ and $\xi$ but for different values inverse temperature parameter ($\beta$).

Figure 9a–d are plot of vibrational entropy against magnetic flux $\left( {\omega_{c} } \right)$ for different values of inverse temperature parameter $(\beta )$, plot of vibrational entropy against AB flux $\left( \xi \right)$ for different values of inverse temperature parameter ($\beta$), plot of vibrational entropy against inverse temperature parameter ($\beta$) for fixed value of $\omega_{c}$ and $\xi$ but for different values of maximum vibrational quantum number ($\lambda$) and plot of vibrational entropy against the maximum vibrational quantum number ($\lambda$), for fixed value of $\omega_{c}$ and $\xi$ but for different values inverse temperature parameter ($\beta$) respectively. In Fig. 9a and d, the vibrational entropy increases exponentially with an increase in magnetic field and maximum vibrational quantum number respectively. In Fig. 9b, the vibrational entropy rises to the peak with maximum turning point at $\xi = 35$ before slopping in a divergence manner with distinct spacing between the spectral curves. In Fig. 9c, the vibrational entropy increases exponentially with an increase in inverse temperature parameter.

**Figure 10:**
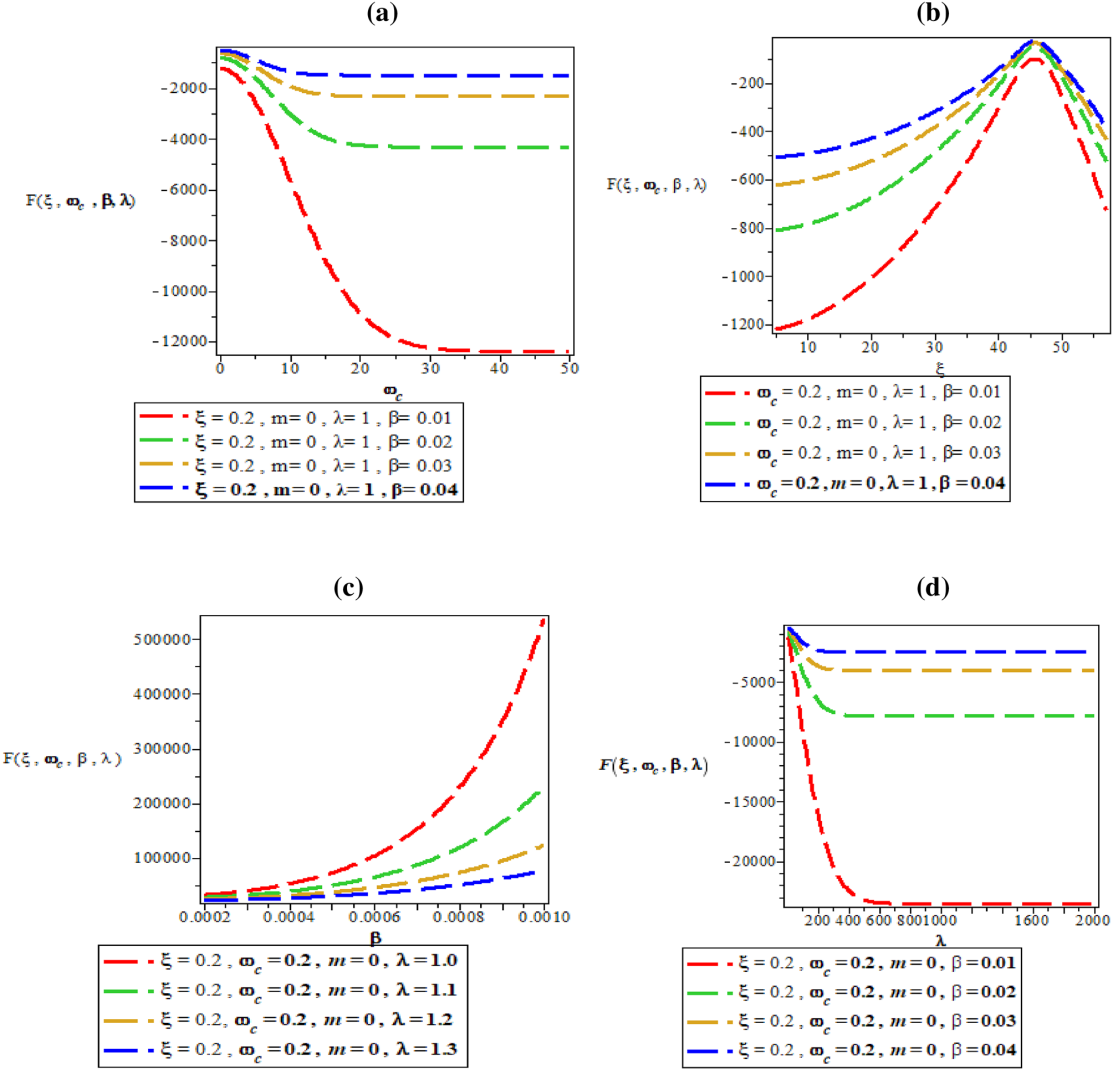
(**a**) Plot of vibrational Free energy against magnetic flux $\left( {\omega_{c} } \right)$ for different values of inverse temperature parameter $(\beta )$. (**b**) Plot of vibrational Free energy against AB flux $\left( \xi \right)$ for different values of inverse temperature parameter ($\beta$). (**c**) Plot of vibrational Free energy against inverse temperature parameter ($\beta$) for fixed value of $\omega_{c}$ and $\xi$ but for different values of maximum vibrational quantum number ($\lambda$). (**d**) Plot of vibrational Free energy against the maximum vibrational quantum number ($\lambda$) for fixed value of $\omega_{c}$ and $\xi$ but for different values inverse temperature parameter ($\beta$).

Figure 10a–d are plot of vibrational Free energy against magnetic flux $\left( {\omega_{c} } \right)$ for different values of inverse temperature parameter $(\beta )$, plot of vibrational Free energy against AB flux $\left( \xi \right)$ for different values of inverse temperature parameter ($\beta$), plot of vibrational Free energy against inverse temperature parameter ($\beta$) for fixed value of $\omega_{c}$ and $\xi$ but for different values of maximum vibrational quantum number ($\lambda$) and plot of vibrational Free energy against the maximum vibrational quantum number ($\lambda$) for fixed value of $\omega_{c}$ and $\xi$ but for different values inverse temperature parameter ($\beta$) respectively. Figure 10a–d has similar explanation to Fig. 9a–d.

**Figure 11:**
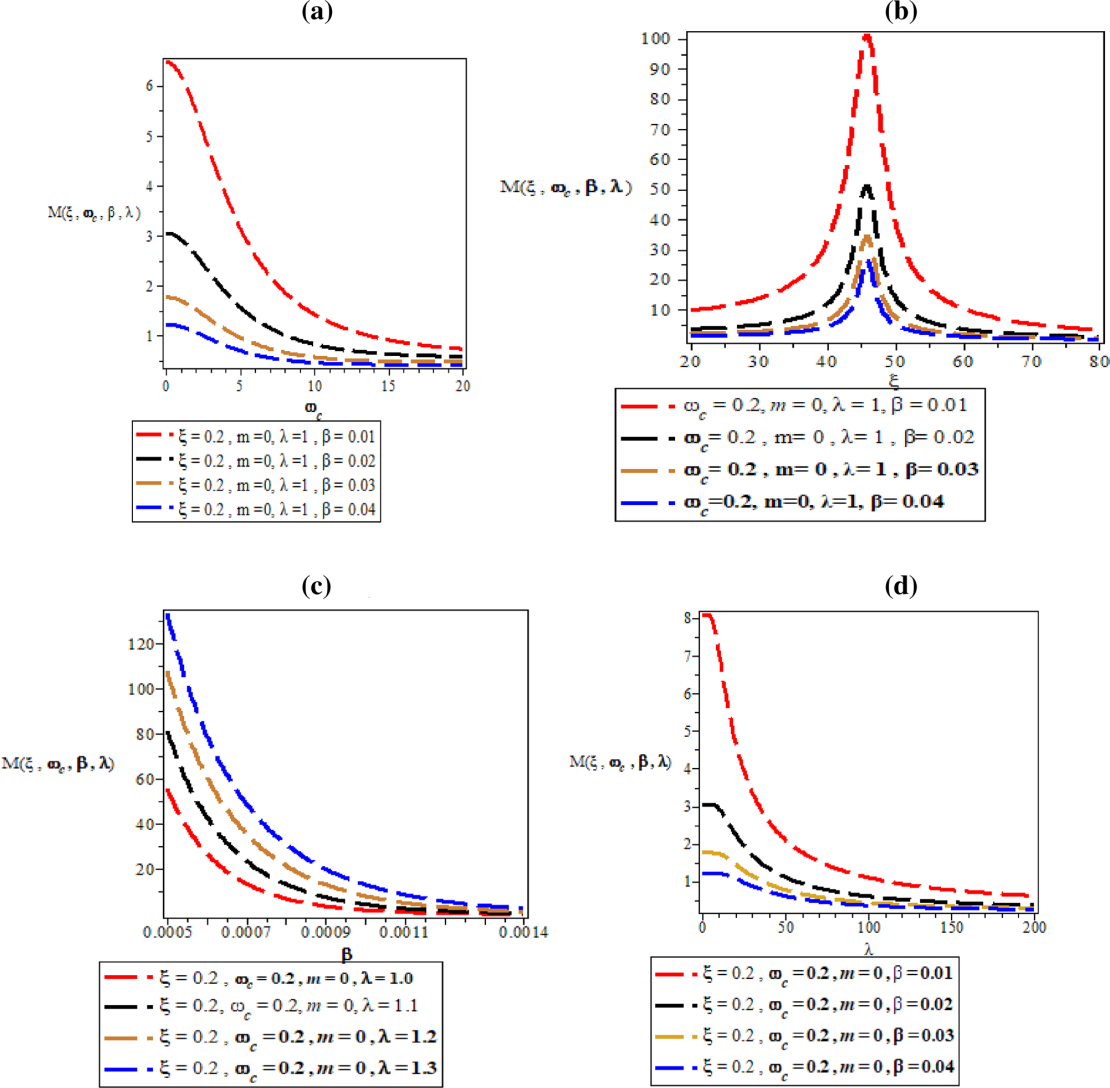
(**a**) Plot of magnetization against magnetic flux $\left( {\omega_{c} } \right)$ for different values of inverse temperature parameter $(\beta )$. (**b**) Plot of magnetization against AB flux $\left( \xi \right)$ for different values of inverse temperature parameter ($\beta$). (**c**) Plot of magnetization against inverse temperature parameter ($\beta$) for fixed value of $\omega_{c}$ and $\xi$ but for different values of maximum vibrational quantum number ($\lambda$). (**d**) Plot of magnetization against the maximum vibrational quantum number ($\lambda$) for fixed value of $\omega_{c}$ and $\xi$ but for different values inverse temperature parameter ($\beta$).

Figure 11a–d. are plot of magnetization against magnetic flux $\left( {\omega_{c} } \right)$ for different values of inverse temperature parameter $(\beta )$, plot of magnetization against AB flux $\left( \xi \right)$ for different values of inverse temperature parameter ($\beta$), plot of magnetization against inverse temperature parameter ($\beta$) for fixed value of $\omega_{c}$ and $\xi$ but for different values of maximum vibrational quantum number ($\lambda$) and plot of magnetization against the maximum vibrational quantum number ($\lambda$) for fixed value of $\omega_{c}$ and $\xi$ but for different values inverse temperature parameter ($\beta$) respectively. In Fig. 11a, c and d, the magnetization increases exponentially with an increase in $\omega_{c}$, $\beta$ and $\lambda$ respectively. However, in Fig. 11b the influence of AB field produces normal distribution curves with distinct peaks corresponding to the values of inverse temperature parameter ($\beta$).

**Figure 12:**
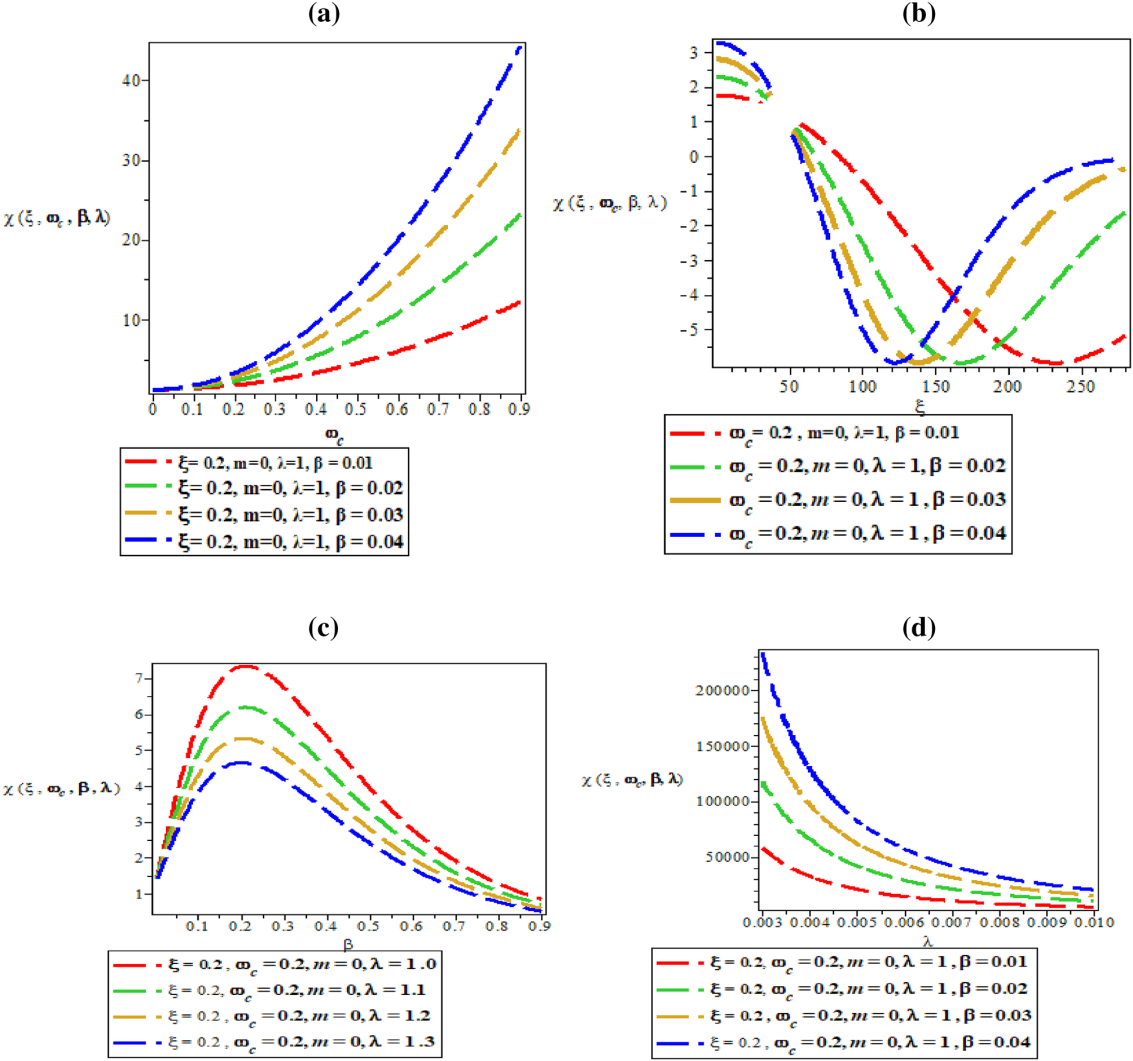
(**a**) Plot of magnetic susceptibility against magnetic flux $\left( {\omega_{c} } \right)$ for different values of inverse temperature parameter $(\beta )$. (**b**) Plot of magnetic susceptibility against AB flux $\left( \xi \right)$ for different values of inverse temperature parameter ($\beta$). (**c**) Plot of magnetic susceptibility against inverse temperature parameter ($\beta$) for fixed value of $\omega_{c}$ and $\xi$ but for different values of maximum vibrational quantum number ($\lambda$). (**d**) Plot of magnetic susceptibility against the maximum vibrational quantum number ($\lambda$) for fixed value of $\omega_{c}$ and $\xi$ but for different values inverse temperature parameter ($\beta$).

Figure 12a–d are plot of magnetic susceptibility against magnetic flux $\left( {\omega_{c} } \right)$ for different values of inverse temperature parameter $(\beta )$, plot of magnetic susceptibility against AB flux $\left( \xi \right)$ for different values of inverse temperature parameter ($\beta$), plot of magnetic susceptibility against inverse temperature parameter ($\beta$) for fixed value of $\omega_{c}$ and $\xi$ but for different values of maximum vibrational quantum number ($\lambda$) and plot of magnetic susceptibility against the maximum vibrational quantum number ($\lambda$) for fixed value of $\omega_{c}$ and $\xi$ but for different values inverse temperature parameter ($\beta$) respectively. In Fig. 12a, the magnetic susceptibility increases monotonically from zero into diverging curves. In Fig. 12b, the magnetic susceptibility produces sinusoidal curves with discontinuity at $\xi = 50$. In Fig. 12c, the magnetic susceptibility rises to attain various local maximum point at precisely $\beta = 0.125\;{\text{K}}^{ - 1}$. Also, in Fig. 12d, the magnetic susceptibility increases exponentially with an increase in maximum vibrational quantum number.

**Figure 13:**
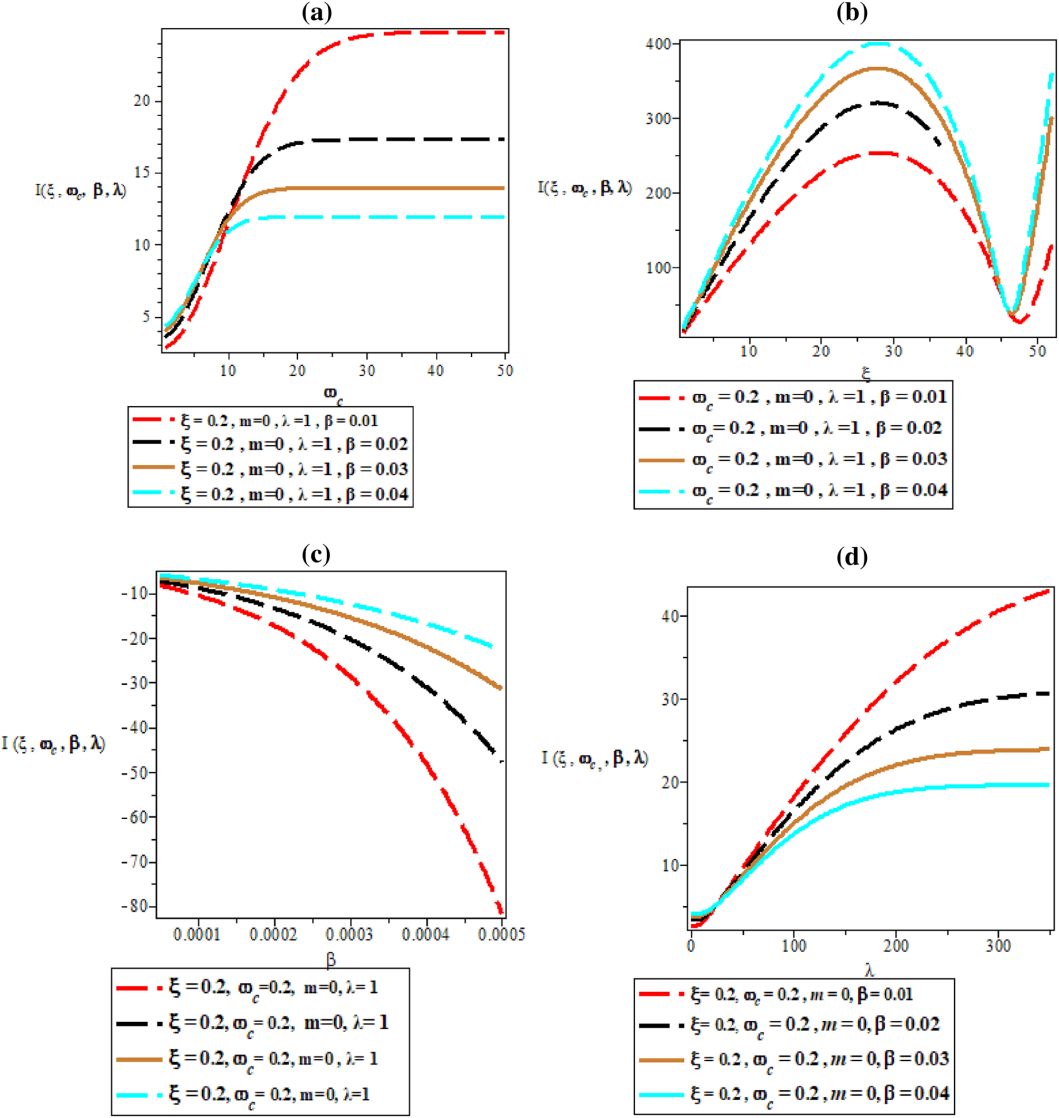
(**a**) Plot of persistent current against magnetic flux $\left( {\omega_{c} } \right)$ for different values of inverse temperature parameter $(\beta )$. (**b**) Plot of persistent current against AB flux $\left( \xi \right)$ for different values of inverse temperature parameter ($\beta$). (**c**) Plot of persistent current against inverse temperature parameter ($\beta$) for fixed value of $\omega_{c}$ and $\xi$ but for different values of maximum vibrational quantum number ($\lambda$). (**d**) Plot of persistent current against the maximum vibrational quantum number ($\lambda$) for fixed value of $\omega_{c}$ and $\xi$ but for different values inverse temperature parameter ($\beta$).

Figure 13a–d are plot of persistent current against magnetic flux $\left( {\omega_{c} } \right)$ for different values of inverse temperature parameter $(\beta )$ **,** plot of persistent current against AB flux $\left( \xi \right)$ for different values of inverse temperature parameter ($\beta$), Fig. 13c plot of persistent current against inverse temperature parameter ($\beta$) for fixed value of $\omega_{c}$ and $\xi$ but for different values of maximum vibrational quantum number ($\lambda$) and plot of persistent current against the maximum vibrational quantum number ($\lambda$) for fixed value of $\omega_{c}$ and $\xi$ but for different values inverse temperature parameter ($\beta$) respectively. In Fig. 13a and d, the persistent current increases asymptotically from the origin with increasing value of magnetic field and maximum upper bound vibrational quantum number. In Fig. 13b, the persistent current rises from the origin to exhibits various maximum points before concaving upward with unique minimum points with maximum at $\xi = 45$. In Fig. 13c, the persistent current increases from the vertical axis in a quantized form before diverging into various spectral curves with increasing value of $\beta$.

**Figure 14:**
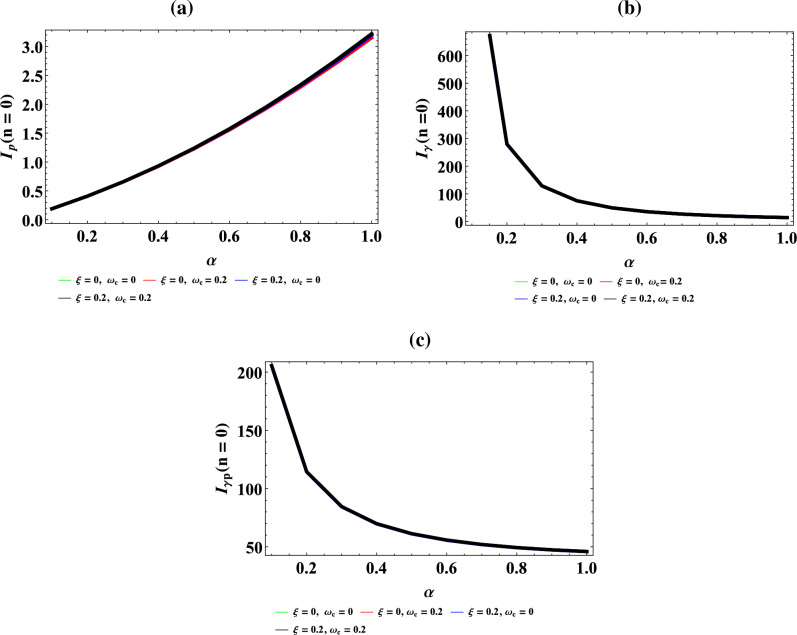
(**a**) The plot of position space Fisher entropy against the screening parameter for $n = 0$. (**b**) The plot of momentum space Fisher entropy against the screening parameter for $n = 0$. (**c**) The plot of product of position and momentum space Fisher entropy against the screening parameter for $n = 0$.

Figure 14a–c are the plot of position space Fisher entropy against the screening parameter for $n = 0$, the plot of momentum space Fisher entropy against the screening parameter for $n = 0$ and the plot of product of position and momentum space Fisher entropy against the screening parameter for $n = 0$ respectively. In Fig. 14a, the position space Fisher entropy increases linearly with an increase in the screening parameter, while the momentum space and its product increases exponentially with an increase in the screening parameter ($\alpha$) as shown in Fig. 14b and c respectively.

**Figure 15:**
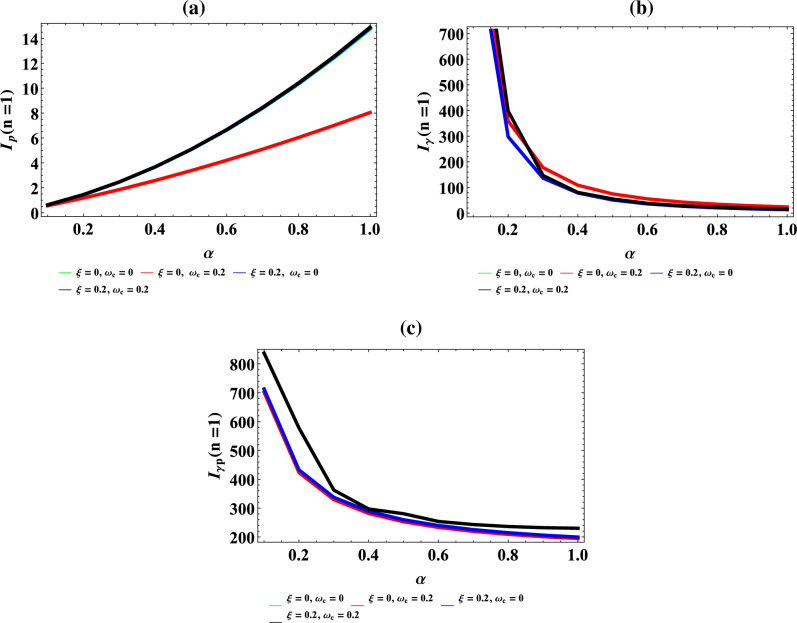
(**a**) The plot of position space Fisher entropy against the screening parameter for $n = 1$ . (**b**) The plot of momentum space Fisher entropy against the screening parameter for $n = 1$. (**c**) The plot of product of position and momentum space Fisher entropy against the screening parameter for $n = 1$.

Figure 15a–c are the plot of position space Fisher entropy against the screening parameter for $n = 1$, the plot of momentum space Fisher entropy against the screening parameter for $n = 1$ and the plot of product of position and momentum space Fisher entropy against the screening parameter for $n = 1$ respectively. Figure 15a–c has the same explanation as Fig. 14a–c.

**Figure 16:**
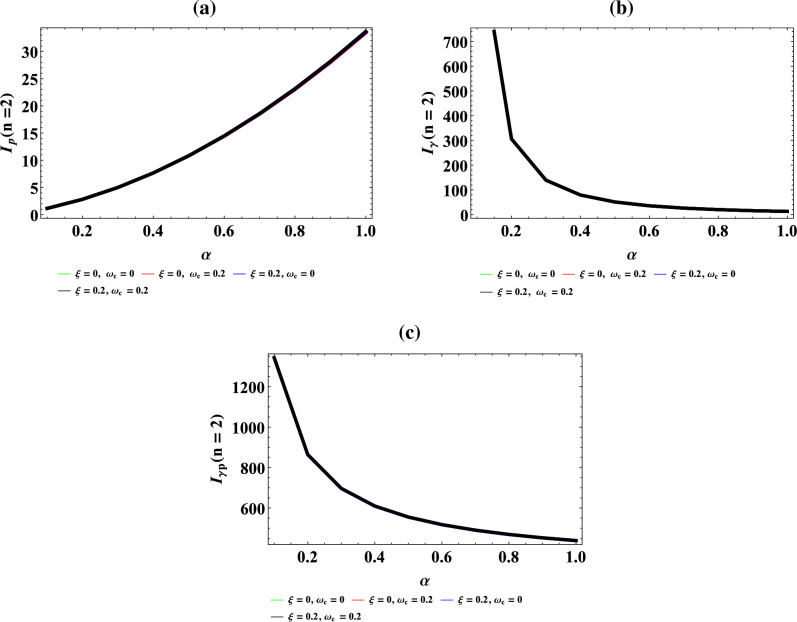
(**a**) The plot of position space Fisher entropy against the screening parameter for $n = 2$. (**b**) The plot of momentum space Fisher entropy against the screening parameter for $n = 2$. (**c**) The plot of product of position and momentum space Fisher entropy against the screening parameter for $n = 2$.

Figure 16a–c are the plot of position space Fisher entropy against the screening parameter for $n = 2$**,** plot of momentum space Fisher entropy against the screening parameter for $n = 2$ and the plot of product of position and momentum space Fisher entropy against the screening parameter for $n = 2$ respectively. Figure 16a–c has the same explanation as Fig. 14a–c.

**Figure 17:**
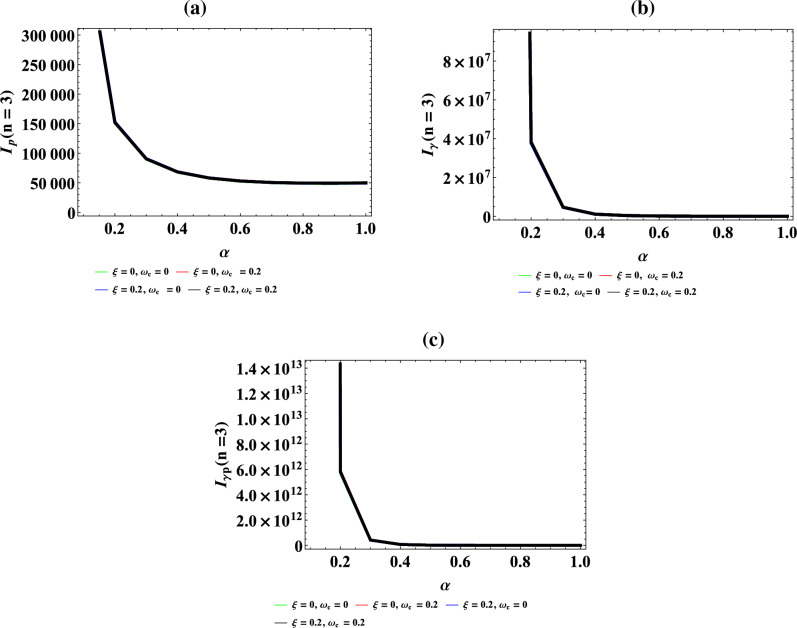
(**a**) The plot of position space Fisher entropy against the screening parameter for $n = 3$. (**b**) The plot of momentum space Fisher entropy against the screening parameter for $n = 3$. (**c**) The plot of product of position and momentum space Fisher entropy against the screening parameter for $n = 3$.

Figure 17a–c are the plot of position space Fisher entropy against the screening parameter for $n = 3$**,** the plot of momentum space Fisher entropy against the screening parameter for $n = 3$ and the plot of product of position and momentum space Fisher entropy against the screening parameter for $n = 3$ respectively. In Fig. 17a, the position space entropy increases exponentially with an increase in the value of $\alpha$. However, in Fig. 17b and c, there is abnormally which makes the plot to decrease with decreasing value of $\alpha$ with respect to momentum space and its products respectively.

Table 1 is the numerical bound state solution for the proposed potential under the influence of AB and Magnetic field for fixed magnetic quantum number but with varying principal quantum number. In Table 1, it can be observed that when both fields are deactivated, i.e., AB and magnetic fields are zero, the energy spectra degenerate; thus, as the number of quantum states increases, the energy spectra decrease. When only the AB field was applied to the quantum system, it resulted in quasi-degeneracy, and the energy spectra decreased with increasing quantum states. When only the magnetic field is activated, the system produces a similar effect, but this time degeneracy is gradually eliminated. When both fields are activated, the combined effects completely eliminates degeneracy from the quantum system's energy spectra. All computation were carried out using the following constant physical parameters: $c_{1} = c_{2} = 1,\sigma_{0} = 0.5,\hbar = \mu = 1,\alpha = 0.2,c = 1$.

**Table 1:** Numerical bound state solution for the proposed potential under the influence of AB and Magnetic field.

$m$	$n$	$\xi = \omega_{c} = 0$	$\xi = 0.2,\omega_{c} = 0$	$\xi = 0,\omega_{c} = 0.2$	$\xi = 0.2,\omega_{c} = 0.2$
0	0	− 0.90133601	− 0.90184811	− 0.89816015	− 0.90059738
	1	− 2.43479386	− 2.43548836	− 2.43180819	− 2.43426196
	2	− 3.97979520	− 3.98031212	− 3.97664018	− 3.97911044
	3	− 5.53577109	− 5.53629036	− 5.53262704	− 5.53511371
− 1	0	− 0.91413788	− 0.90952922	− 0.90133601	− 0.89865265
	1	− 2.44783595	− 2.44320564	− 2.43497386	− 2.43228286
	2	− 3.99271706	− 3.98806518	− 3.97979520	− 3.97709679
	3	− 5.54875214	− 5.54407895	− 5.53577109	− 5.33065400
1	0	− 0.91413788	− 0.91977099	− 0.92058755	− 0.92814565
	1	− 2.44783595	− 2.45349560	− 2.43497386	− 2.43228286
	2	− 3.99271706	− 3.99840302	− 3.99932855	− 4.00696761
	3	− 5.54875214	− 5.55446409	− 5.55544480	− 5.56312395

Tables 2, 3, 4 and 5 are the numerical computation for position, momentum, and products Fisher entropy under the influence of AB and magnetic field for $n = 0$ to $n = 3$, respectively. In these Tables, it is clear that our results obey Heisenberg uncertainty principles in which there is uncertainty in the simultaneous measurement of the position and momentum of quantum mechanical particles. The numerical results also show that as the values for position Fisher entropy increase, the momentum values decrease with an increase in screening parameters. This trend holds for all quantum states in the absence of both magnetic and AB fields, only magnetic fields, only AB fields, and the combined influence of both magnetic and AB fields.

**Table 2:** Numerical values for position, momentum and products Fisher entropy under the influence of AB and Magnetic field for $n = 0$.

α	$\xi = \omega_{c} = 0$			$\xi = 0,\omega_{c} = 0.2$			$\xi = 0.2,\omega_{c} = 0$			$\xi = \omega_{c} = 0.2$		
	$I\left( p \right)$	$I\left( \gamma \right)$	$I\left( p \right)I\left( \gamma \right) \ge 16$	$I\left( p \right)$	$I\left( \gamma \right)$	$I\left( p \right)I\left( \gamma \right) \ge 16$	$I\left( p \right)$	$I\left( \gamma \right)$	$I\left( p \right)I\left( \gamma \right) \ge 16$		$I\left( \gamma \right)$	$I\left( p \right)I\left( \gamma \right) \ge 16$
0.1	0.190558	1076.98	205.22715	0.18993	1082.04	205.51186	0.190594	1076.82	205.23544	0.190269	1080.82	205.64654
0.2	0.408783	279.481	114.24708	0.40751	280.724	114.39671	0.409079	279.323	114.26517	0.409170	280.026	114.57824
0.3	0.654802	128.777	84.32344	0.65285	129.319	84.42643	0.655806	128.319	84.42643	0.656610	128.794	84.56743
0.4	0.928695	75.0143	69.66541	0.92606	75.3134	69.74488	0.931095	74.8544	69.44516	0.933376	74.8739	69.88550
0.5	1.230510	49.6664	61.11500	1.22718	49.8541	61.17995	1.235230	49.5054	61.15056	1.239610	49.4658	61.31830
0.6	1.560270	35.5478	55.62019	1.55622	35.7754	55.67439	1.568470	35.4856	55.65809	1.575560	35.4210	55.80791
0.7	1.917600	27.0456	51.87238	1.91319	27.1373	51.91881	1.931050	26.8823	51.91107	1.941490	26.8070	52.04552
0.8	2.303560	21.3661	49.21809	2.29805	21.4347	49.25801	2.323190	21.2017	49.25558	2.337600	21.1221	49.37502
0.9	2.717000	17.4064	47.29319	2.71076	17.4594	47.32319	2.745080	17.2411	47.32819	2.764100	17.1605	47.43334
1.0	3.158240	14.5274	45.88102	3.15125	14.5692	45.91119	3.196900	14.3610	45.91068	3.221150	14.2811	46.00157

**Table 3:** Numerical values for position, momentum and products Fisher entropy under the influence of AB and Magnetic field for $n = 1$.

α	$\xi = \omega_{c} = 0$			$\xi = 0,\omega_{c} = 0.2$			$\xi = 0.2,\omega_{c} = 0$			$\xi = \omega_{c} = 0.2$		
	$I\left( p \right)$	$I\left( \gamma \right)$	$I\left( p \right)I\left( \gamma \right) \ge 16$	$I\left( p \right)$	$I\left( \gamma \right)$	$I\left( p \right)I\left( \gamma \right) \ge 16$	$I\left( p \right)$	$I\left( \gamma \right)$	$I\left( p \right)I\left( \gamma \right) \ge 16$	$I\left( p \right)$	$I\left( \gamma \right)$	$I\left( p \right)I\left( \gamma \right) \ge 16$
0.1	0.62863	1130.46	710.63542	0.55935	1254.06	701.45469	0.62873	1130.31	22,024.4819	0.62782	1134.05	711.97927
0.2	1.45190	297.293	431.63971	1.17879	358.994	423.17854	1.45274	297.154	431.68750	1.45277	297.754	432.56808
0.3	2.46684	136.589	336.94321	1.85599	177.313	329.09115	2.46965	136.462	337.01338	2.47242	136.602	337.73752
0.4	3.67059	78.6762	288.78807	2.58877	108.710	281.42519	3.67714	78.5601	288.87649	3.68441	78.579	289.51725
0.5	5.06060	51.2734	259.47417	3.37509	74.8220	252.53098	5.07318	51.1675	259.58194	5.08663	51.1478	260.16993
0.6	6.63467	36.1291	239.70864	4.21306	55.3381	233.14274	6.65603	36.0321	239.83074	6.67729	35.9999	240.38177
0.7	8.39101	26.8697	225.46392	5.10091	42.9848	219.26159	8.42432	26.7804	225.60666	8.45494	26.7452	226.12906
0.8	10.3282	20.7901	214.72431	6.03699	34.5987	208.87201	10.3770	20.7077	214.88380	10.4155	20.6732	215.32171
0.9	12.4451	16.5809	206.35096	7.01975	28.6112	200.84347	12.5134	16.5045	206.52741	12.5672	16.4721	207.00818
1.0	14.7410	13.5443	199.65653	8.04777	24.1672	194.49207	14.8331	13.4731	199.84784	14.9005	13.4432	200.31040

**Table 4:** Numerical values for position, momentum and products Fisher entropy under the influence of AB and Magnetic field for $n = 2$.

$\alpha$	$\xi = \omega_{c} = 0$			$\xi = 0,\omega_{c} = 0.2$			$\xi = 0.2,\omega_{c} = 0$			$\xi = \omega_{c} = 0.2$		
	$I\left( p \right)$	$I\left( \gamma \right)$	$I\left( p \right)I\left( \gamma \right) \ge 16$	$I\left( p \right)$	$I\left( \gamma \right)$	$I\left( p \right)I\left( \gamma \right) \ge 16$	$I\left( p \right)$	$I\left( \gamma \right)$	$I\left( p \right)I\left( \gamma \right) \ge 16$	$I\left( p \right)$	$I\left( \gamma \right)$	$I\left( p \right)I\left( \gamma \right) \ge 16$
0.1	1.14998	1166.80	1341.79666	1.14706	1171.26	1343.50549	1.15015	1166.65	1341.8225	1.14871	1170.14	1344.15152
0.2	2.82094	306.016	863.25278	2.81553	306.951	864.22975	2.82231	305.895	863.33052	2.82264	306.416	864.90206
0.3	4.99644	139.322	696.11401	4.9890	139.672	696.82360	5.00091	139.220	696.22669	5.00608	139.338	697.53178
0.4	7.66194	79.4702	608.89590	7.65295	79.6384	609.46869	7.67219	79.3840	609.04913	7.68512	79.4040	610.22927
0.5	10.8054	51.3227	554.56230	10.7953	51.4154	555.04467	10.8248	51.2484	554.75368	10.8482	51.2393	555.85417
0.6	14.4173	35.8696	517.14278	14.4066	35.9256	517.56575	14.4498	35.8049	517.37364	14.4863	35.7871	518.42267
0.7	18.4903	26.4822	489.66382	18.4792	26.5183	490.03697	18.5402	26.4252	489.92849	18.5923	26.4057	490.94269
0.8	23.0187	20.3557	488.56175	23.0077	20.3802	468.90153	23.0910	20.3052	468.86737	23.1610	20.2864	483.23110
0.9	27.9985	16.1376	451.82859	27.9879	16.1547	452.13612	28.0986	16.0923	452.17110	28.1887	16.0750	453.13335
1.0	33.4271	13.1099	438.22593	33.4170	13.1224	438.51124	23.5606	13.0691	307.91584	33.6728	13.0535	439.54789

**Table 5:** Numerical values for position, momentum and products Fisher entropy under the influence of AB and Magnetic field for $n = 3$.

$\alpha$	$\xi = \omega_{c} = 0$			$\xi = 0,\omega_{c} = 0.2$			$\xi = 0.2,\omega_{c} = 0$			$\xi = \omega_{c} = 0.2$ = 0.2		
	$I\left( p \right)$	$I\left( \gamma \right)$	$$\begin{gathered} I\left( p \right)I\left( \gamma \right) \hfill \\ \ge 16 \hfill \\ \end{gathered}$$	$I\left( p \right)$	$I\left( \gamma \right)$	$$\begin{gathered} I\left( p \right)I\left( \gamma \right) \hfill \\ \ge 16 \hfill \\ \end{gathered}$$	$I\left( p \right)$	$I\left( \gamma \right)$	$$\begin{gathered} I\left( p \right)I\left( \gamma \right) \hfill \\ \ge 16 \hfill \\ \end{gathered}$$	$I\left( p \right)$	$I\left( \gamma \right)$	$$\begin{gathered} I\left( p \right)I\left( \gamma \right) \hfill \\ \ge 16 \hfill \\ \end{gathered}$$
0.1	469,346.0	1.48039 × 10^9^	6.94815 × 10^14^	472,860.0	1.50117 × 10^9^	7.09843 × 10^14^	469,419.0	1.48026 × 10^9^	6.94862 × 10^14^	474,011.0	1.50179 × 10^9^	7.11864 × 10^14^
0.2	151,773.0	3.77707 × 10^7^	5.73257 × 10^12^	152,716.0	3.82080sx10^7^	5.83497 × 10^12^	151,868.0	3.77674 × 10^7^	5.73565 × 10^12^	153,480.0	3.82755 × 10^7^	5.87452 × 10^12^
0.3	90,290.3	4.62718 × 10^6^	4.17789 × 10^11^	90,783.9	4.67359 × 10^6^	4.24286 × 10^11^	90,410.2	4.62759 × 10^6^	4.18381 × 10^11^	91,454.0	4.68925 × 10^6^	4.28850 × 10^11^
0.4	68,133.3	1.09200 × 10^6^	7.44015 × 10^10^	68,471.1	1.10177 × 10^6^	7.54394 × 10^10^	68,280.9	1.09245 × 10^6^	7.45934 × 10^10^	69,124.9	1.10725 × 10^6^	7.65385 × 10^10^
0.5	57,979.8	3.69366 × 10^5^	2.14137 × 10^10^	58,245.2	3.72367 × 10^5^	2.16885 × 10^10^	58,158.7	3.69684 × 10^5^	2.15003 × 10^10^	58,916.9	3.74827 × 10^5^	2.20836 × 10^10^
0.6	52,863.4	1.56622 × 10^5^	8.27957 × 10^9^	53,089.4	1.57793 × 10^5^	8.37714 × 10^9^	53,077.8	1.56843 × 10^5^	8.32488 × 10^9^	53,799.1	1.59088 × 10^5^	8.55879 × 10^9^
0.7	50,305.6	7.74768 × 10^4^	3.89751 × 10^9^	50,508.0	7.80152 × 10^4^	3.94039 × 10^9^	50,599.8	7.76354 × 10^4^	3.92834 × 10^9^	51,269.5	7.87785 × 10^4^	4.03893 × 10^9^
0.8	49,228.6	4.28314 × 10^4^	2.10853 × 10^9^	49,416.1	4.31100 × 10^4^	2.13033 × 10^9^	49,527.2	4.29491 × 10^4^	2.12715 × 10^9^	50,240.3	4.35986 × 10^4^	2.19041 × 10^9^
0.9	49,096.1	2.57418 × 10^4^	1.26382 × 10^9^	49,273.7	2.58994 × 10^4^	1.27616 × 10^9^	49,443.8	2.58320 × 10^4^	1.27723 × 10^9^	50,170.3	2.62324 × 10^4^	1.31609 × 10^9^
1.0	49,613.6	1.65069 × 10^4^	8.18967 × 10^8^	49,784.7	1.66025 × 10^4^	8.26550 × 10^8^	50,015.4	1.65781 × 10^4^	8.29160 × 10^8^	50,762.3	1.68409 × 10^4^	8.54883 × 10^8^

Correspondingly, our numerical results in all quantum states satisfy the 2D local Fisher uncertainty product inequality expressed as ($I\left( \rho \right)I\left( \gamma \right) \ge 16$ as shown in Tables 2, 3, 4 and 5 for all quantum states. All our results clearly show that as the quantum state increases, the values of position increases, while that of momentum and product values decrease. The Fisher product values in all quantum states clearly show the localization of the quantum mechanical particles both in the absence and presence of magnetic and AB fields. Finally, the numerical results from their product indicate that the particle is more localized when the combined effect of AB and magnetic fields on the entropy than the absence of both fields, as shown by ( ($I\left( \rho \right)I\left( \gamma \right) \ge 16$).

## Conclusion

In this work, we study analytical solutions, thermomagnetic properties, and its effect on Fisher information entropy with Schioberg plus Manning-Rosen potential using the Nikiforov-Uvarov functional analysis and Supersymmetric quantum mechanics methods. We obtained the energy equation in a closed and compact form both in NUFA and SUSYQM and applied the solution to study partition function and other thermomagnetic properties.

The trend of thermomagnetic plots is in excellent agreement with the work of existing literature. Using the normalized wave function, we obtained the wave function and probability density plots and applied them to study Fisher information entropy in position and momentum spaces. The numerical results show that the combined impact of the magnetic and AB flux fields completely removes the degeneracy on the energy spectra and that increasing the screening parameter increases the position of Fisher entropy while decreasing its momentum, satisfying the 2D local Fisher uncertainty product condition. It also causes both localization and delocalization of quantum particles. Meanwhile, as the quantum state increases under the combined influence of magnetic and AB fields, the results of Fisher entropies and the product increase. Finally, the proposed potential reduces to Schioberg and Manning-Rosen potential as special cases. The wave function and probability density plots were obtained using Maple 10,0 software, while the position and momentum Fisher entropies were obtained using a well-designed Mathematica program.

## Data Availability

The data available in this manuscript are obtained using maple and Mathematica programme from the resulting energy eigen equation.
